# Robust Spatial Modeling of Thermodynamic Parameters in a Full-Scale Reverse Osmosis Membrane Channel

**DOI:** 10.1021/acsomega.0c04412

**Published:** 2021-05-07

**Authors:** Afra Alkatheeri, Ramis Rafay, Emad Alhseinat, Ahmad Safieh, Fadi Alnaimat

**Affiliations:** † Department of Chemical Engineering, Khalifa University of Science and Technology, P.O. Box 127788, Abu Dhabi, United Arab Emirates; ‡ Center for Advanced Membranes and Water Technologies, P.O. Box 127788, Abu Dhabi, United Arab Emirates; § Research & Development Department, Dubai Electricity & Water Authority (DEWA), P.O. Box 564, Dubai, United Arab Emirates; ∥ Mechanical Engineering Department, United Arab Emirates University (UAEU), P.O. Box 15551, Al Ain, United Arab Emirates

## Abstract

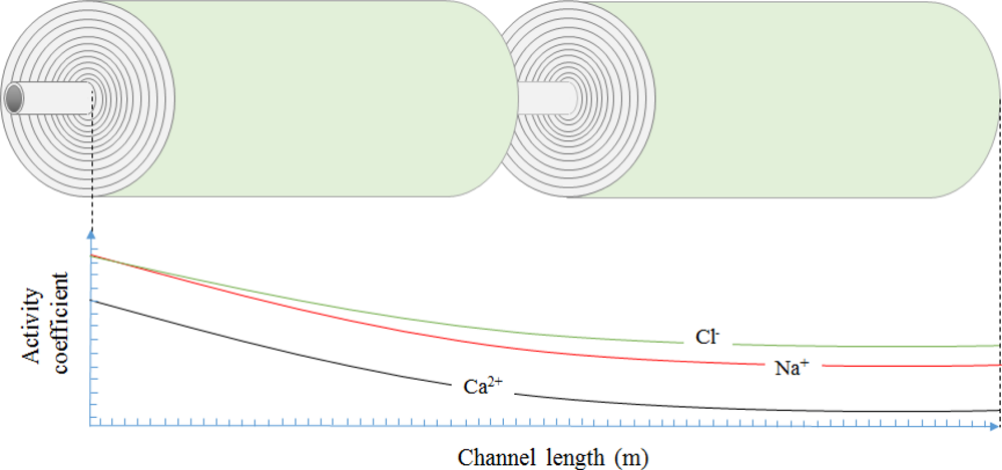

Full-scale reverse osmosis (RO) units usually consist of a set of pressure vessels holding up to six (1 m long) membrane modules in series. Since process parameters and water composition change substantially along the filtration channel in full-scale RO units, relevant thermodynamic parameters such as the ion activities and the osmotic coefficient change as well. Understanding these changes will lead to more accurate fouling prediction and to improvement in process and equipment designs. In this article, a rigorous thermodynamic model for RO concentrates in a full-scale module is developed and presented, which is capable of accounting for such changes. The change in concentrate composition due to permeation of water and ions is predicted locally in the membrane filtration channel. The local ionic composition is used to calculate the local activity coefficient and osmotic coefficient along the membrane channel through the Pitzer model for each modeled anion and cation. The approach developed was validated against related literature data, showing that Pitzer coefficient predictions were satisfactory. The spatial variation model was verified experimentally. It was found under the modeled conditions of high recovery that individual solute activity coefficients could be diminished up to 65%, in our case for sulfate, from their initial value from the membrane inlet to the outlet, and the water osmotic coefficient increased 3% as concentrate salinity increased from the membrane inlet to the outlet. Modeled at moderate recovery, the sulfate still achieved a statistically significant drop of 34% and an opposing trend of a decrease of 0.5% for the osmotic coefficient. These variations in internal water chemistry along the channel can significantly impact predicted recovery, fouling propensity, and permeate quality. Fouling prediction with our approach was also assessed through a theoretical fouling index to demonstrate the significance of ion activity over concentration-based calculations. Additionally, data from a pilot plant RO filtration channel was used to carry out a sensitivity analysis to show the capability of the developed model.

## 1 Introduction

Reverse osmosis (RO) membranes are a class of pressure-driven thin-film composite membranes. They target the removal of dissolved species via semipermeable membranes. To achieve drinking water standards using seawater as feed, Loeb and Sourirajan, 60 years ago, developed the first batch of RO membranes.^1^ Since then, RO processes have been highly optimized for energy efficiency and solute separation and are currently responsible for half of the global clean water production.^2^

Full-scale RO units usually consist of a set of pressure vessels holding up to six (1 m long) membrane modules in series. A “black box” approach, considering solely system inlet and outlet averages, has been found to be sufficient for describing the behavior of a small segment of RO membranes, utilizing fundamental transport theories.^3,4^ For long membrane filtration channels, the use of this approach to describe the performance has been criticized due to the substantial variation in parameters along the channel.^3,5−8^ A newer practice in modeling involves a finite difference approach (through discretization) to obtain realistic values for process parameters in full-scale systems.^4^

Thermodynamic parameters dictate the fouling potential of RO systems. The mechanisms regulating the flow behavior of long-channel RO were evaluated by Song et al. under numerous run conditions.^3,5^ They found that the thermodynamic equilibrium enforces a significant restriction, namely, thermodynamic restriction, on full-scale RO performance especially on the permeate flux near the end of the channel where there is an increase in osmotic pressure caused by the salt buildup approaching the applied hydraulic pressure. They also stressed on the difference in flow progression under thermodynamic restriction and mass transfer mechanisms. Operating at high pressures, recovery is solely dependent on the salt concentration and transmembrane pressure. In his paper, he simulates around 3000 ppm brackish feed and 35 bar applied pressure and finds thermodynamic restriction to play a role once more than three 1 m modules are involved. In a three-stage simulation, the first stage was unaffected by the restriction but contribution was evident in the second and third stage.^9^ Due to the development of commercial RO membranes with high permeability and low inherent resistance, the traditional use of operating parameters such as permeate flux decline is no longer considered suitable as a fouling indicator in commercial seawater RO systems.^10^ Zhu et al. [2, 3] published a number of articles considering thermodynamic restriction in the optimization of the specific energy consumption for single- and multistage RO systems. They stated that due to these highly permeable/high-rejection membranes, plants can theoretically operate as close to the limit of thermodynamic restriction to achieve energy savings as the cost of multistage operation for low-salinity brackish water would require justification.^11^ Therefore, thermodynamic restriction and its impact on permeation are essential for understanding the operation of a full-scale RO.^9^ Tay and Song reported that the thermodynamic equilibrium can be the dominant mechanism controlling the permeate flux under common run conditions.^12^ When the system is operated close to the limits of the thermodynamic equilibrium, the flux becomes unaffected by the changes in membrane resistance. In the connection of fouling to the permeate flux, an experimental study for calcium carbonate fouling by Tzotzi et al. found inconsistencies between observed fouling and the permeate flux decline as fouling growth continued; a delayed response was found in permeation.^13^ An optical technique reported by Lyster et al. reported the same finding.^14,15^ Thus, systems such as these cannot be accurately described by the drop in permeate flux. Reliable prediction of feed water thermodynamic properties considering this restriction along with a well-studied fouling index could provide valuable insights into the nature of the observed fouling behavior inside a long membrane filtration channel.

Spatial modeling of concentration and flow behavior in membrane modules is imperative as direct measurements from the inner segments of a module are still impossible.^15^ Many simple one-dimensional to more complex three-dimensional spatial transport models were developed to describe solution behavior in a membrane channel but a limited number consider the compositional changes along long filtration channels.^2^ Hoek et al. monitored the long-term performance of a large pilot plant of 21 membrane elements. Through the database built, a one-dimensional model was developed and reasonably predicted plant performance for more than 4 months of operation while modeling concentration and pressure drop per element.^4^ Bernales et al. modeled the flow in both the transverse and axial directions while accounting for the changes in the concentration boundary layer. This successfully estimated the progression of the concentration profile with every step/segment and its effect on permeation under their described set of conditions.^16^ The transport and concentration polarization results of a three-dimensional CFD analysis carried out by Li et al. were extracted and introduced into a one-dimensional spatial model that was able to predict concentration polarization for a full-scale spiral module.^2,17^ These studies opted to use generalized global chemical parameters which were sufficient for the intended objective of modeling overall trends; however, an added benefit is expected in extending the models to include properties of individual species in solution. Karabelas et al. developed a two-dimensional spatial model that simulated single-ion behavior for a brackish-water RO crossflow test cell. Their model suggests a change in flow and concentration behavior along the width and the length as the section simulated of the membrane approaches the permeate tube in a spiral wound module. This creates a more accurate depiction of behavior in specific areas of the membrane sheet.^18,19^ More recent work by Song et al. described a 2-D model assuming a shear flow pattern with a nonuniform velocity profile in the *y*-direction (along the height). The concentration polarization profile was found to be affected by the choice of the flow pattern depending on the channel height.^20^ Nevertheless, both Karabelas et al. and Song et al. found through their simulation that under reasonable conditions, a narrow channel (i.e., 7 × 10^–4^ m in the typical range of height for industrial applications) with negligible permeate pressure, a 1-D model satisfies the prediction requirement for an RO long filtration channel. As important as it is to accurately depict the spatial geometry of spiral wound modules, there are still benefits for the more spatially reductionist one-dimensional models that can reliably describe solution behavior in RO particularly since thermodynamic ideality is taken as an assumption in most of the aforementioned models.^4,8,9,16,17^

Cohen et al. considers the point of thermodynamic restriction the minimum required pressure to utilize the entire axial length of a train membrane module. In their calculations, the assumption made is the proportional relationship between the osmotic pressure and concentration which in reality consistently deviate along the channel due to changes imposed by the growing ionic strength.^21^ Since thermodynamic restrictions can govern the performance of RO membranes, considering that thermodynamic nonideality is key to predicting observed behavior. Key studies aim to address this in various ways. Sheikholeslami explain limitations in their previous work and others in the disregard of solution nonidealities when assessing thermodynamic behavior.^22^ Jang et al. recognized this shortcoming in transport models and developed the Merten and Londsdale model salt transport coefficients.^23^ These coefficients, unique to each salt, represent the salt concentration gradient from the retentate to the permeate side. Considering thermodynamic nonideality through Pitzer-calculated activity was seen to significantly impact the salt transport.

Activity coefficient calculations also play a significant role in evaluating scale propensity. In this study, solute activity coefficients refer to the solute unsymmetrical rational activity coefficients that achieve ideality at infinite dilution.^24^ Thiel and Lienhard calculated the saturation index as a fouling measure for produced water through an activity model to aid in the selection of the treatment route for the water.^25^ Karabelas et al. accounted for activities as well, assessing fouling tendency of calcium sulfate through an external program “PHREEQC v.3”. Additionally, they correlate solution supersaturation to an experimentally measured scale-mass density component that allows it to simulate the scale deposition rate.^18,19^ PHREEQC and Minteq are among publicly available water chemistry programs that provide a variety of parameters including activity coefficients.^19^ PHREEQC has a coupling feature that allows developers to easily integrate it into their models. Although accessibility to thermodynamic models gives tremendous benefits, discrepancies may occur as assumptions vary with each applied system.^26^ The presented model incorporates the Pitzer activity model with the RO transport model to accurately simulate retentate solution thermodynamics for each individual ion in the concentrate stream.

In this article, we build on the work of Alhseinat and Sheikholeslami that takes changes in local ion concentration and local flow behavior in the axial direction into consideration.^27^ The model is based on a series of differential mass and momentum balances and applied to predict local thermodynamics parameters in full-scale RO along the axial length of the membrane filtration channel. The main novelty is in combining the spatial variation prediction in composition and local thermodynamic behavior. In addition, the model also provides key thermodynamic parameters for each individual ion which demonstrates applicability to true sea water RO systems and displays model flexibility. Its application for fouling prediction was investigated through a theoretical inorganic fouling index. Plant data provided from a pilot plant ran by Dubai Electricity and Water Authority was used to conduct a study to view the effect of operating conditions on process performance.

## 2 Results and Discussion

### 2.1 Model Validation

#### 2.1.1 Pitzer Model

To determine the accuracy of the thermodynamic models, the program was applied to a specific seawater composition to determine activity and osmotic coefficients for key ionic species and the results were compared against relevant published experimental data for seawater, and the results are presented in Tables 1 and 2.

**Table 1:** Single-Ion Activity Coefficients Calculated Using Our Program and by Others^a^

reference	*I* (mol/Kg)	m Na+	γ Na+	m K+	γ K+	m Ca2+	γ Ca2+	m Mg2+	γ Mg2+	m Cl–	γ Cl^–^	m SO2^–^	γ SO4^–^
Hamrouni and Dhahbi^a^^28^	0.73	0.493	0.640	0.017	0.599	0.011	0.190	0.056	0.206	0.576	0.691	0.029	0.121
Millero and Schreiber^a^^29^	0.70		0.69		0.615		0.228		0.255		0.628		0.085
this work^a^	0.73	0.493	0.646	0.017	0.518	0.011	0.197	0.056	0.2038	0.576	0.5179	0.029	0.097
Hajbi et al.^b^^30^	1.05	0.651	0.619	0.017	0.557	0.017	0.183	0.09	0.198	0.816	0.691	0.047	0.085
this work^b^	1.04		0.631		0.475		0.188		0.201		0.585		0.078
Hajbi et al.^c^^30^	1.58	0.83	0.636	0.027	0.54	0.025	0.227	0.133	0.265	1.397	0.673	0.061	0.06
this work^c^	1.57	0.83	0.647	0.027	0.566	0.025	0.226	0.133	0.257	1.4	0.5313	0.061	0.055

a “m *i*” and “γ *i*” denote the molality and activity coefficient for ionic species *i*, respectively.

b Superscripts a, b, and c represent sections evaluating the same input compositions.

**Table 2:** Osmotic Coefficients as Calculated Using the Developed Program and Some Literature Values^a^

reference	NaCl molality (mol/Kg)	ϕosmotic
Hajbi et al.a^30^	0.65	0.973
Hamer and We^a^^31^	0.6	0.923
Hamer and We^a^^31^	0.7	0.926
this work^a^	0.65	0.916
Hajbi et al.^b^^30^	0.83	0.956
Hamer and We^b^^31^	0.8	0.929
this work^b^	0.83	0.946

a Superscripts a and b represent sections evaluating similar input compositions.

Hamrouni and Dhahbi^28^ and Hajbi et al.^30^ used the Pitzer model for the activity coefficient calculations, while Millero and Schreiber^29^ used the ion pairing model to estimate the activity coefficients. When comparing predictions made by this study and others, the developed model is able to provide reasonable estimations of the activity coefficients and osmotic coefficient at various salinity levels and water compositions. Variation in activity coefficient prediction (compared to Pitzer-based models) are greater in ions K^+^, Cl^–^, and SO_4_^2–^ ranging from 5 to 25%, while ions Na^+^, Ca^+^, and Mg^2+^ were successfully estimated with 1–4% error. The osmotic coefficient calculation against other models was found to have 1–6% error. The difference between the current work and the others is due to the assumptions used by each author. In this work, for ease of computation, whole ions are assumed to be free in the water,^29^ and thus, all species are assumed to be fully ionized.

#### 2.1.2 Validation of Spatial Variation

**Figure 1:**
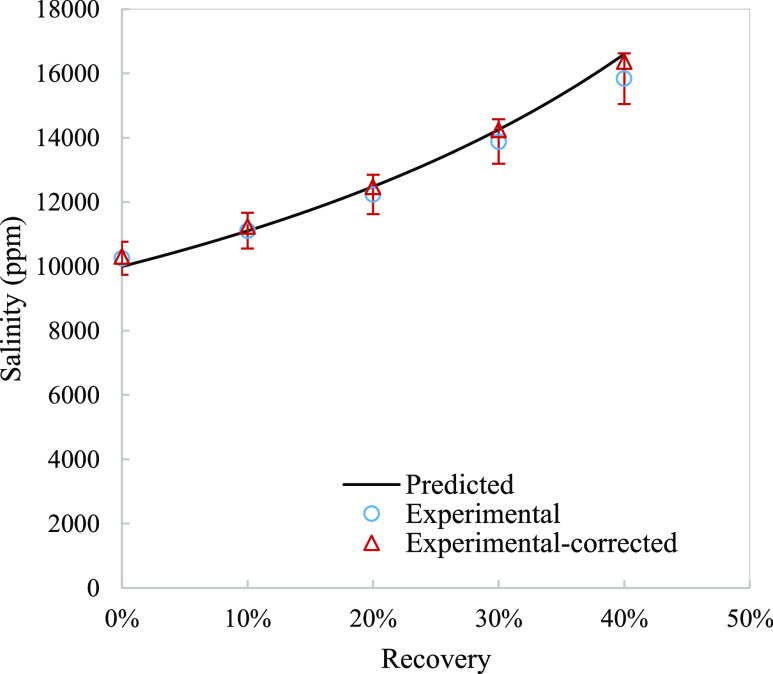
Measured results compared to the model simulated for concentrate salinity before and after correcting for water activity at different recoveries. Error bars represent ±5%.

**Figure 2:**
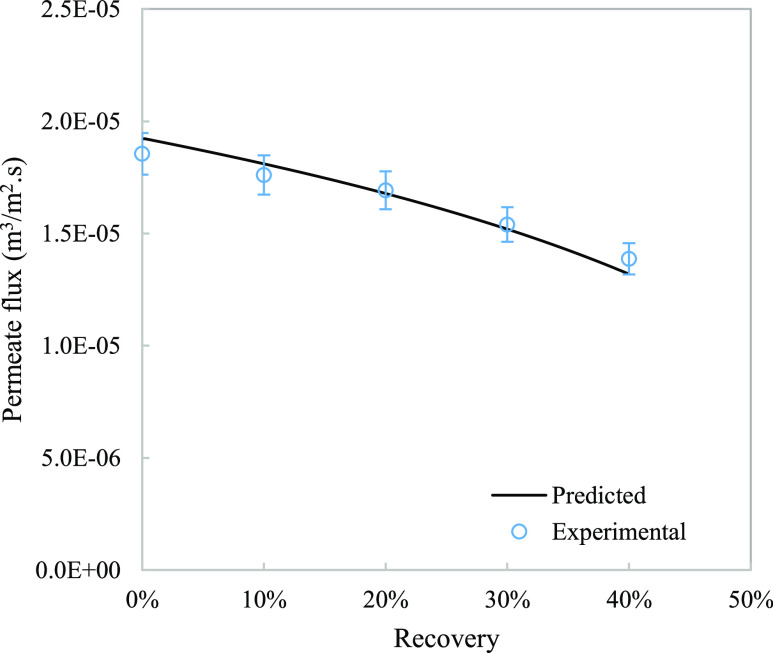
Measured results compared to the model simulated for permeate flux at different recoveries. Error bars represent ±5%.

Following the validation procedure described in Section 4.3, the experimental data were plotted with the model prediction for increase in recovery (Figures 1 and 2).

The model predicted the progression of salinity and permeate flux well as both parameters show an error within 5% (Figures 1 and 2). Correcting for the water activity improved the model comparison especially for the highest recovery value.

### 2.2 Variation in System Parameters throughout the Channel Length

The cases modeled here are single-stage high recovery systems which differ from plant-installed RO systems that have usually low recovery for a single stage or high recovery for a multistage operation. These are available in Section 2.3. The purpose of modeling high recovery is to showcase the internal behavior as solution concentration creates an osmotic pressure that approaches the applied pressure.

#### 2.2.1 Impact of Osmotic Coefficient Prediction

**Figure 3:**
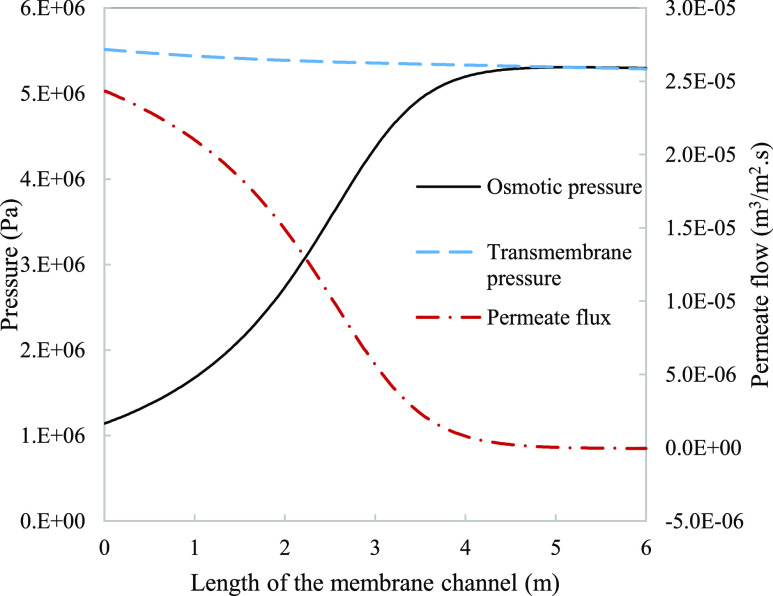
Changes in system parameters as it approaches thermodynamic equilibrium leads to a subsequent halt of permeation. The difference between transmembrane pressure (dashed line) and osmotic pressure (solid line) is the driving force for permeate flow (dashed-dot line) throughout the channel length. Parameters used to model these values were taken from Table 3.

**Figure 4:**
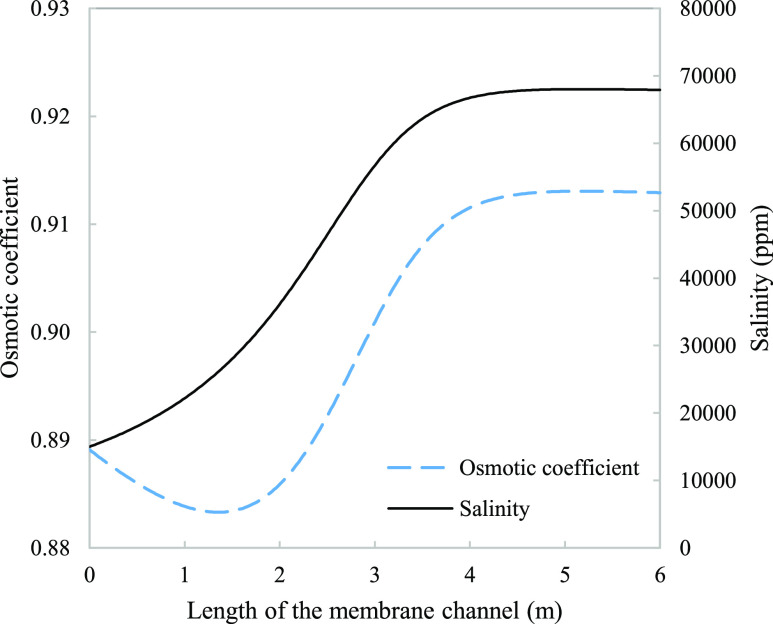
Osmotic coefficient (dashed line) variation along a 6 m membrane channel, and this value tapers off as the system approaches equilibrium. Salinity curve (solid line) indicates the effect of solute concentration/ionic strength on osmotic coefficient prediction. Parameters used to model these values were taken from Table 3.

**Figure 5:**
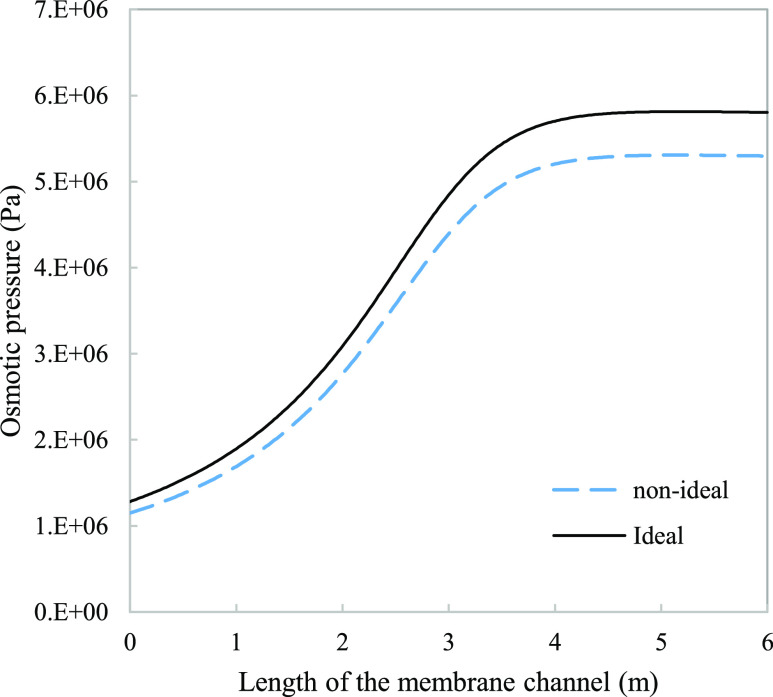
Examining the effect of assuming solution ideality on osmotic pressure modeling. The nonideal (dashed line) curve represents variation in the osmotic coefficient using Pitzer, whereas the ideal (solid line) curve employs the assumption of an ideal thermodynamic behavior. Parameters used to model these values were taken from Table 3.

Pressure is the main driving force of solute–solvent separation in RO membranes. For solvent permeation to occur, the selected operating pressure must always be sufficient to overcome the osmotic pressure. At the same time, excess operating pressure increases energy requirements (increases operating expenses) and reduces the overall plant efficiency. This makes the accurate calculation of the osmotic pressure important for RO system operators. Faults in this parameter estimation usually stem from nonrepresentative assumptions and require continuous reassessment as computational abilities develop. For this reason, the osmotic coefficient (φ) is accounted for in our approach through eqs S.6–S.8 and the Pitzer model. φ depends on solution composition and is particularly important for high-salinity feed. In this study, the local φ is predicted at increasing points (*n* = 300) for the length of the membrane along with the changes in the brine composition. The effect of its prediction on the system performance was examined through Figures 3–5.

Osmotic pressure influences permeation through eq S.6. When the osmotic pressure is equal to the transmembrane pressure, the system reaches a thermodynamic equilibrium where there is no driving force for mass transfer and therefore no effective rejection of salt (Figure 3). The negative flux observed is also an indicative to the degree of polarization near the end of the channel against the friction losses of the applied pressure, causing the minor reversal of the flux direction. The simulated osmotic coefficient follows a nonmonotonic behavior along the channel length. In Figure 4, there is a minor dip in the osmotic coefficient from 0 to 2 m along the membrane channel, after which the coefficient increases until the curve starts to plateau beyond the 4 m point. A similar trend in the osmotic coefficient was observed elsewhere.^32^ Following an increase in salinity from 15,000 to 67,915 ppm, the osmotic coefficient increases by 3% between the feed (0 m) to the final modeled length (6 m), which corresponds to the expected finding from refs (5)(33), and (34). Sahu and Juvekar interpreted this phenomenon as the combined effect of electrostatic and nonelectrostatic interactions contributing to the osmotic coefficient. The nonelectrostatic forces show a linear increase with molality, while electrostatic forces exhibit an exponential decay. At low salinities, the electrostatic forces dominate osmotic coefficient behavior, whereas the nonelectrostatic forces take control at high salinities, corresponding to the trend depicted in Figure 4. The loss of dependency on solution concentration as it increases is due to the shielding effect of the central ion through counterions.^35^ Moreover, the osmotic coefficient corresponds to solution entropy which increases with the weakening of the field of interaction as the number of ions present elevate.^36^Figure 5 evaluates the common assumption of solution ideality (φ = 1 in eq S.8) and compares it with our Pitzer incorporated model. The calculated osmotic pressure along the membrane channel length is overestimated when assuming ideal solutions, up to a maximum percentage deviation of 9% at the membrane outlet (for the described system and may grow larger for a seawater feed at 35,000 ppm).

#### 2.2.2 Impact of Activity Coefficient Prediction

**Figure 6:**
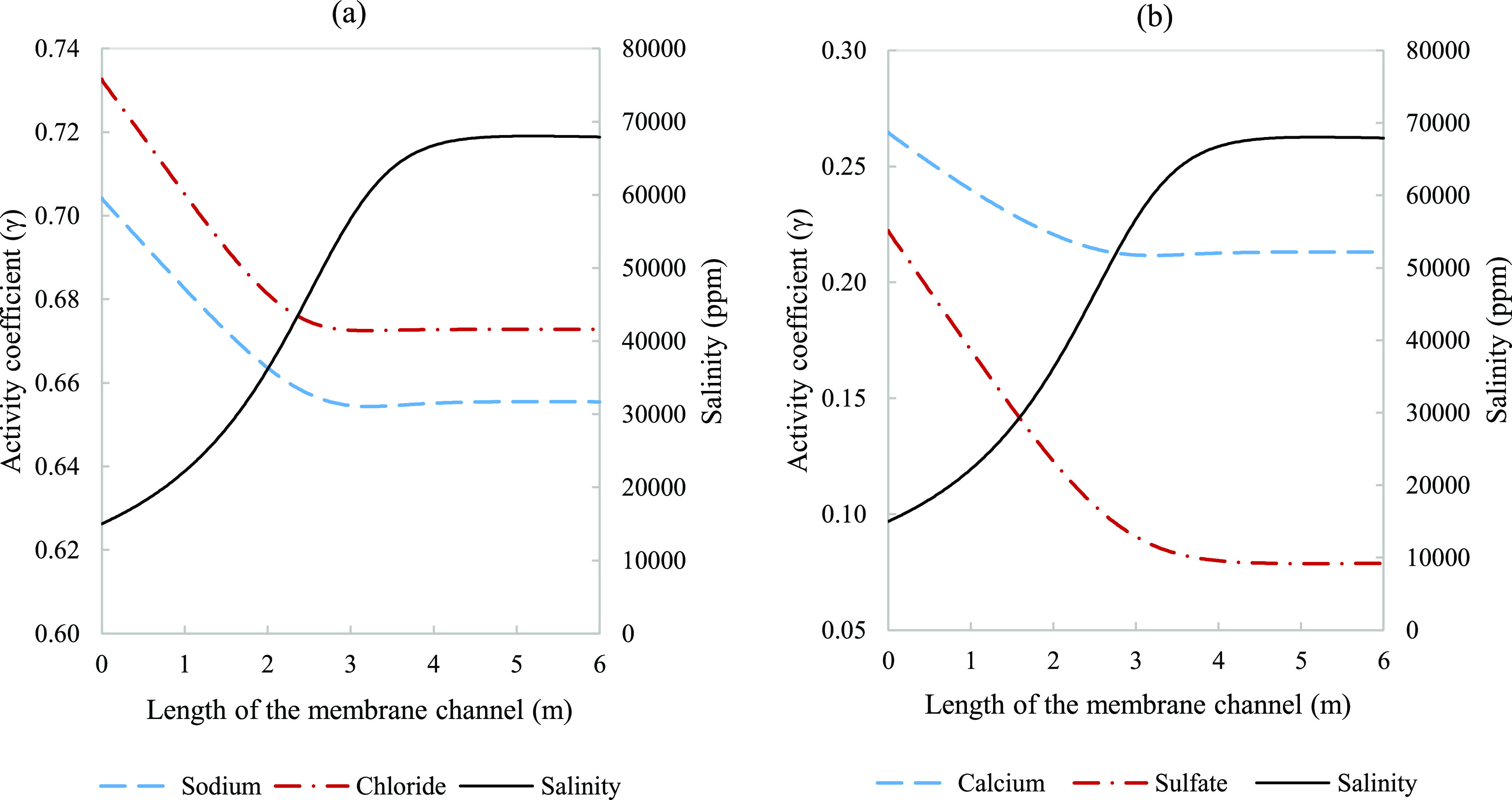
Effect of salinity (solid line) on activity coefficients for (a) monovalent ions sodium (dash line) and chloride (dash dot line) and (b) divalent ions calcium (dash line) and sulfate (dash dot line). Parameters used to model these values were taken from Table 3.

**Figure 7:**
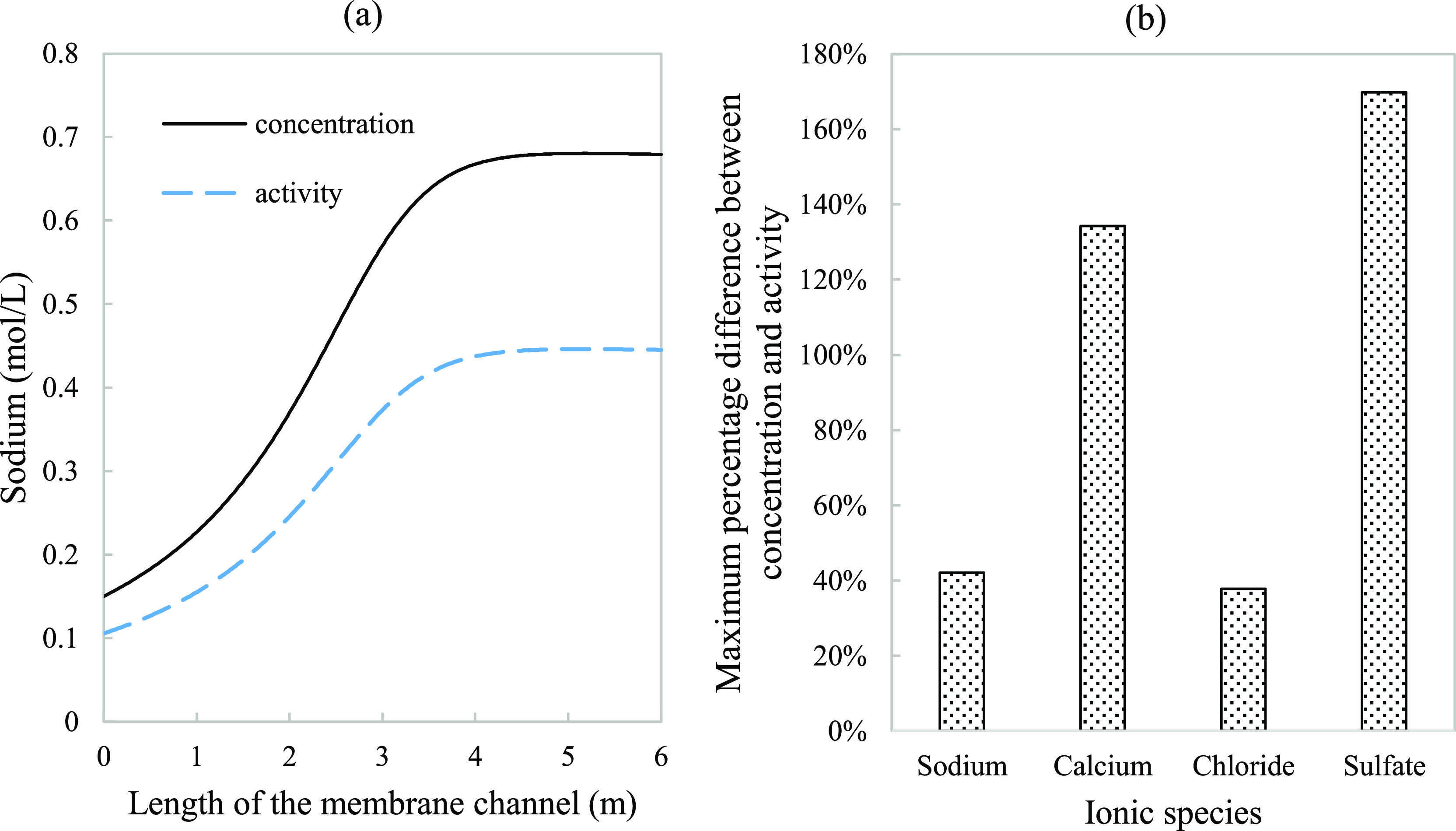
Concentration (solid line) and activity (dash line) were modeled along the length of a 6 m channel membrane assembly: (a) sodium was provided as an example to demonstrate the changes in activity and concentration throughout the channel. (b) Maximum percentage difference at the 6 m point between the two curves. Parameters used to model these values were taken from Table 3.

As shown in Figure 5, high-salinity solutions do not conform to thermodynamic ideality. The deviation from ideality or representation of “real behavior” in the solution composition is taken into consideration through the activity coefficient (γ). Since the activity coefficient for a given ion is dependent on both its individual ionic charge (and valence) and the solution ionic strength, it is expected to change dramatically in the concentrate channel for different ions. Figure 6 shows how activity coefficients for monovalent and divalent ion pairs changes through the channel length; it also indicates that ions of different valencies are affected differently. By considering the maximum relative deviation, the extent to which ion concentrations and activities are not congruent in the high ionic strength environments found in RO membranes is explored in Figure 7.

The calculated activity coefficients for sulfate, calcium, sodium, and chloride respectively, decreased along the membrane channel length by 65, 19, 7, and 8%, due to the increase in brine salinity (Figure 6). Nonmonotonic trends in activity coefficients, as observed for calcium and sodium in Figure 6, have been encountered elsewhere.^32^ Exploring the effect of the feed type, the difference in the activity coefficient from 15,000 to 30,000 ppm (brackish water operation) was found to be 14% for calcium and 35% for sulfate. Comparing it to seawater operation from 30,000 to 60,000 ppm, the two ions exhibit opposing trends where it decreases for calcium to 7% and an increase for sulfate to 40%. The monovalent ions dropped from 5 and 6% for sodium and chloride, respectively, to 3% for both ions. This shows the variation with application and the dependency on the initial conditions. The differences in activity noticed are due to the binary and ternary interaction parameters in the Pitzer model that makes the activity of each ionic species sensitive to the solution composition. Therefore, accounting for the contribution of all dominant and precipitating species is needed for accurate prediction. Figure 7 shows the extent of deviation between calculated ion concentrations and activities. Due to the effect of increasing salinities, these differences were at their maximum at the end of the channel. For RO systems, these differences in sodium and chloride prediction, specifically, impact the simulated membrane rejection and expected permeate quality.^23^ This emphasizes the conclusion made by several authors^22,23,37^ regarding the impact of solution nonideality in performance prediction.

#### 2.2.3 Impact of Considering Activity on Fouling (Scaling) Prediction

**Figure 8:**
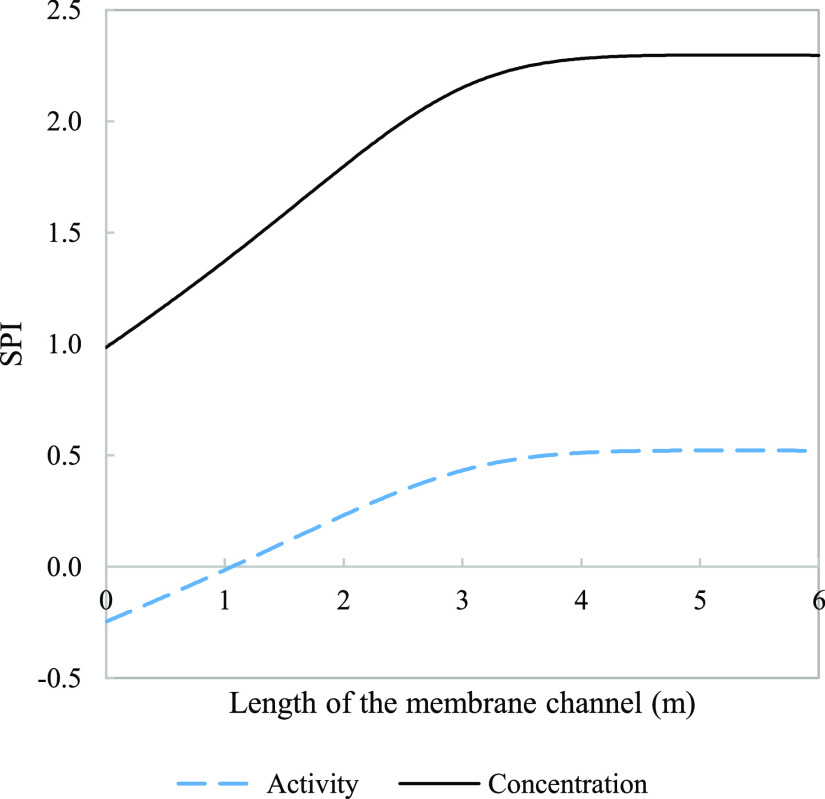
Local SPI considering activity (dashed line) and concentration (solid line) for calcium sulfate modeled along the length of a 6 m channel membrane assembly. Parameters used to model these values were taken from Table 3.

**Figure 9:**
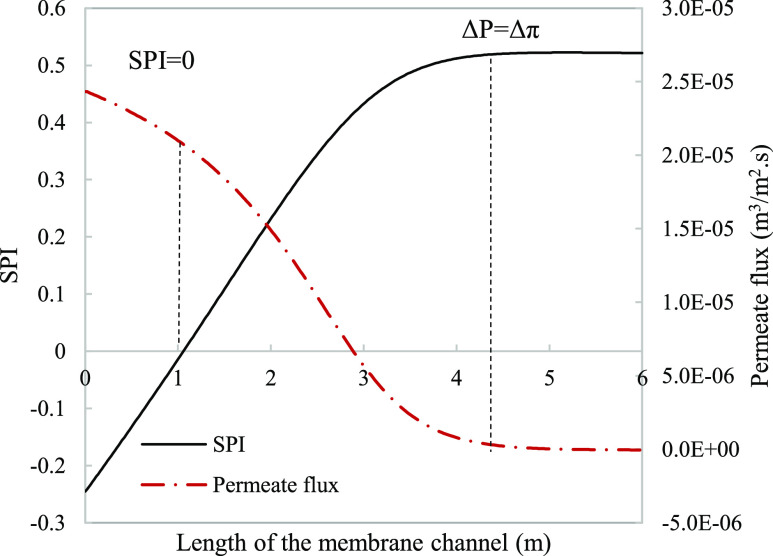
Difference between fouling predicting using a fouling index SPI and relying on the permeate flux decline. SPI (dashed-dot line) and permeate flux (solid line) were plotted along with the dashed line representing the start of fouling conditions and the point of thermodynamic equilibrium. Parameters used to model these values were taken from Table 3.

Continuing on earlier work carried out by Alhseinat and Sheikholeslami,^27^ activity calculations were incorporated into the existing fouling predicting model. The scaling potential index (SPI) regarded as a powerful theoretically based index was used along with the Pitzer model (eq 1).
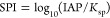
1IAP represents the ion activity product, and *K*_sp_ represents the thermodynamic solubility constant. The system is considered supersaturated when it is positive and the alternative if negative (further described in Supporting Information section 3). Figures 8 and 9 assess the problem arising from neglecting activity and depending on permeate flux decline for evaluating fouling.

The local SPI predicted by the concentration-based calculations indicates that the solution has potential for gypsum scale from the point of entrance, while the activity-predicted SPI shows a delayed initiation of fouling tendency to 1.1 m (Figure 8). Inaccuracies in fouling prediction can result in the inefficient design of a treatment regime. The use of pretreatment chemicals such as acids to lower water pH below the solubility range and antiscalants including phosphonates, phosphates, and polycarboxylates is among the common techniques employed to address scaling in RO systems.^38^ Excessive use of these reagents can cause a negative environmental impact when discharged as brine and has been linked to causing biofouling.^39^ The change in chemical requirement is difficult to evaluate due to the outsourcing from chemical producing companies that treat this as a case-by-case scenario without a clear and systematic protocol reported.^18^

Figure 9 shows the great error that comes from associating the loss in permeation at the point of thermodynamic equilibrium to the presence of fouling. Supersaturated conditions are expected much earlier when considering local SPI rather than permeation loss to assess fouling. This leads to late scheduling of membrane cleaning, resulting in nonrecoverable damage of areas in the membrane.^40^ An interpretation of the underlying causes behind this event is that the unutilized membrane area past the thermodynamic equilibrium point compensates for the deactivation of the membrane area under fouling attack which maintains the average permeate flux at a constant level before severe fouling takes place. An experimental study carried out for CaCO_3_ corroborates this as the ratio of surface blockage was continuously higher than the flux decline.^13^ This form of irreversible fouling leads to unit replacement which accounts for 19% of the overall plant operation and maintenance cost.^39^

### 2.3 Commonly Applied Systems

**Figure 10:**
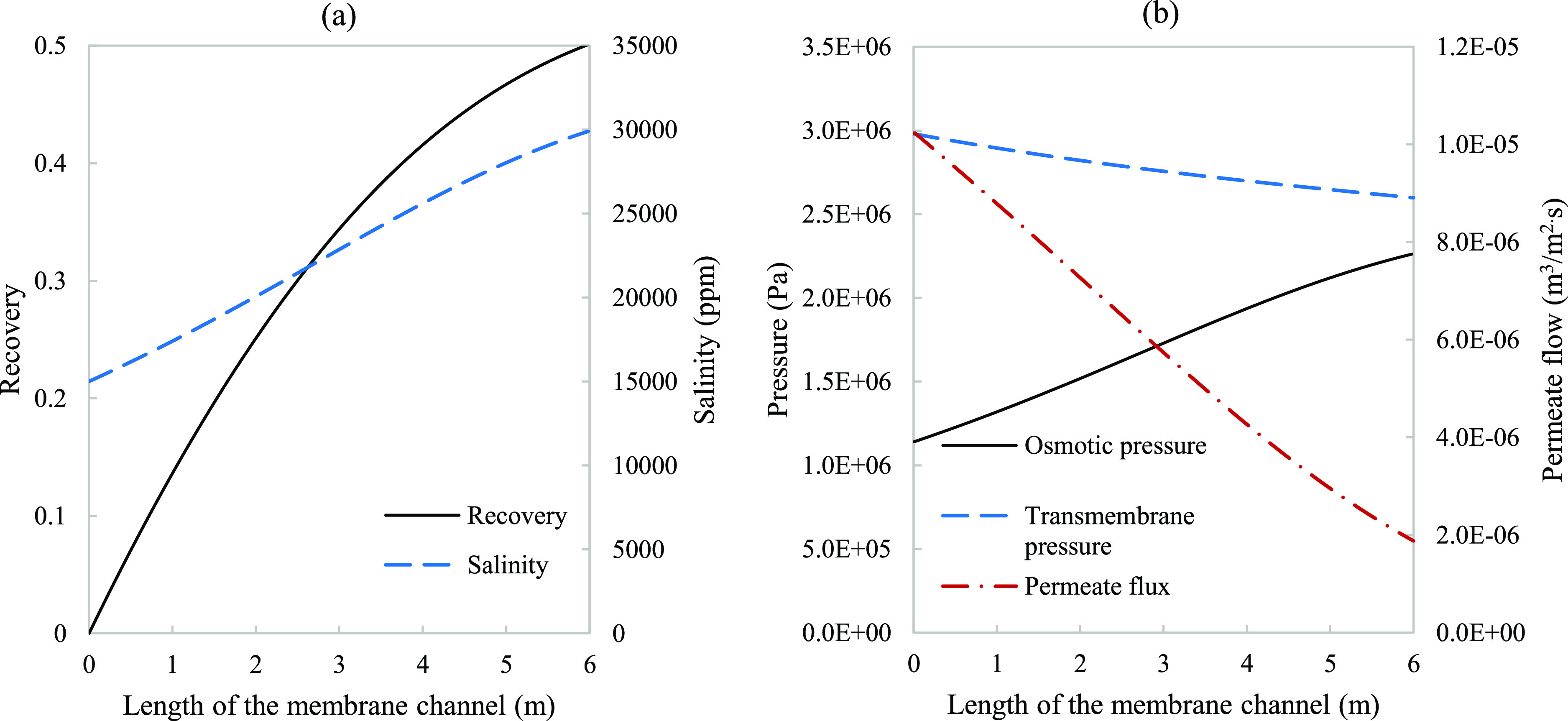
Modeling variation along a 6 m membrane channel of a single-stage system with 50% recovery for (a) recovery (dashed line) along with a salinity curve (solid line) and (b) osmotic pressure (solid line), transmembrane pressure (dashed line), and permeate flux (dash dot line). Parameters used to model these values were taken from Table 3 except for pressure which is 29.8 bar.

**Figure 11:**
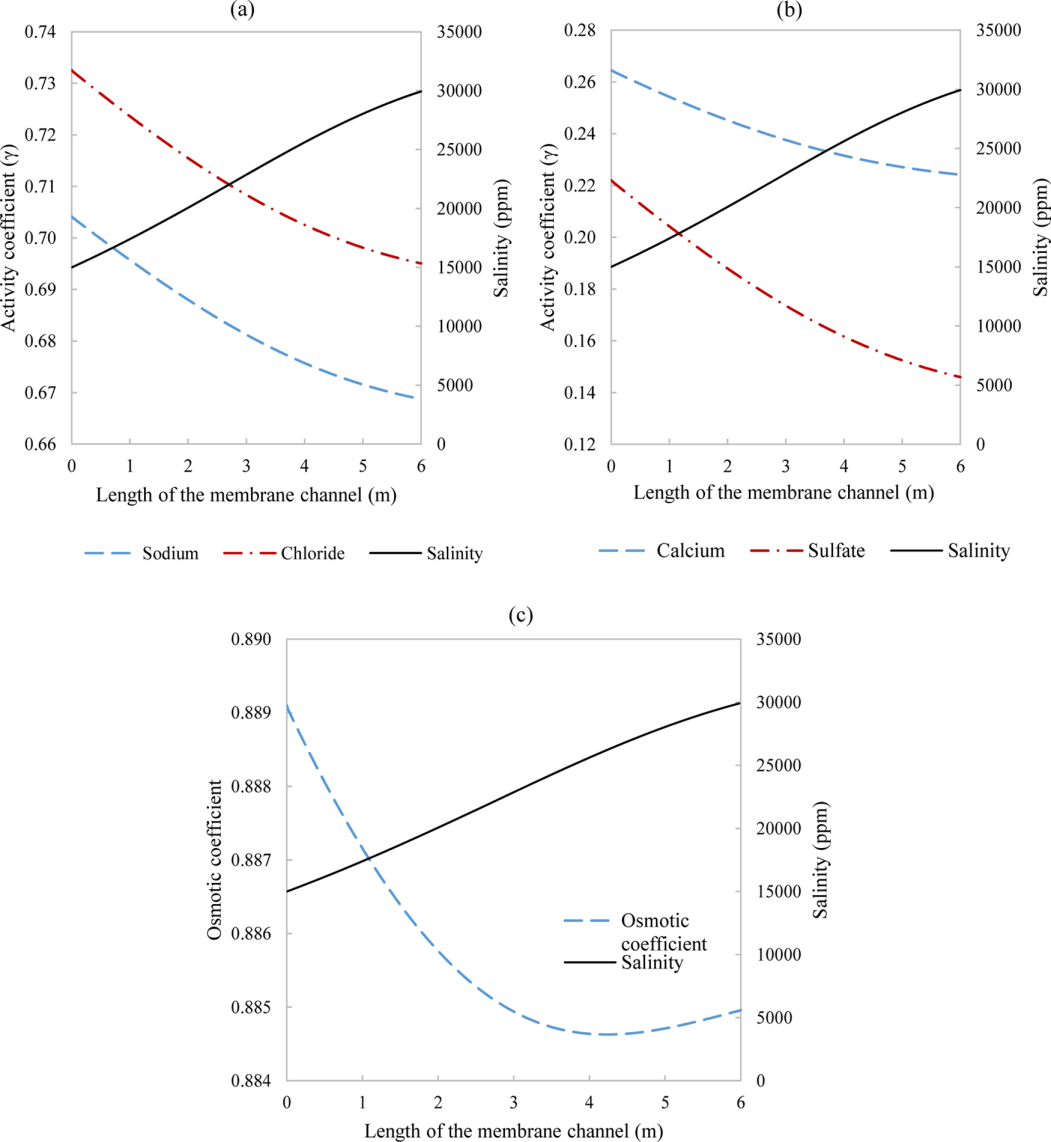
Effect of salinity (solid line) on (a) activity coefficients for monovalent ions sodium (dash line) and chloride (dash dot line), (b) activity coefficients for divalent ions calcium (dash line) and sulfate (dash dot line), and (c) osmotic coefficient for a single-stage system with 50% recovery. Parameters used to model these values were taken from Table 3 except for pressure which is 29.8 bar.

**Figure 12:**
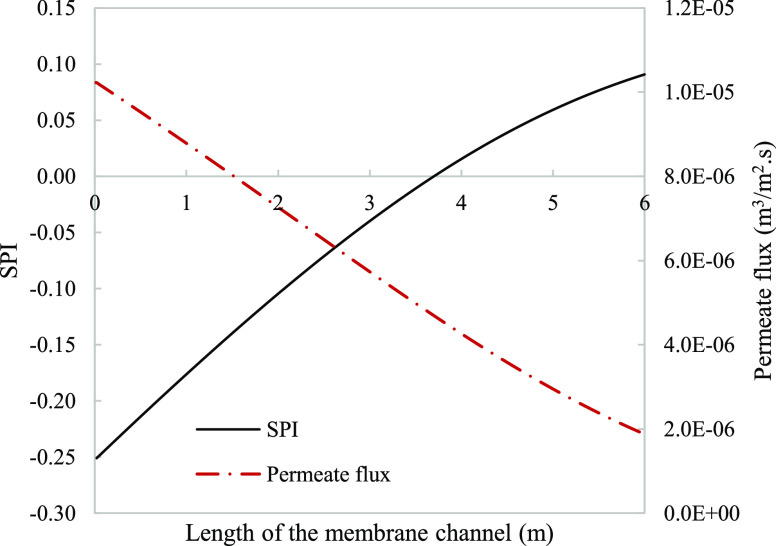
SPI (dashed-dot line) and permeate flux (solid line) were plotted along a 6 m channel for a single-stage system with 50% recovery. Parameters used to model these values were taken from Table 3 except for pressure which is 29.8 bar.

**Figure 13:**
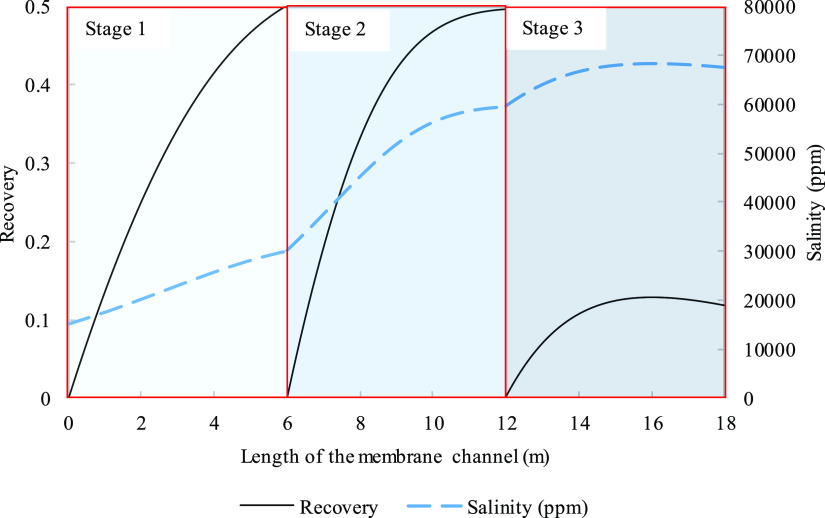
Recovery (solid line) of a three-stage system and variation along three 6 m membrane channels along with a salinity curve (dashed line). Parameters used to model these values were taken from Table 3 except for pressure. Pressures for the three stages were 29.8, 50, and 57 bar in the order of appearance.

**Figure 14:**
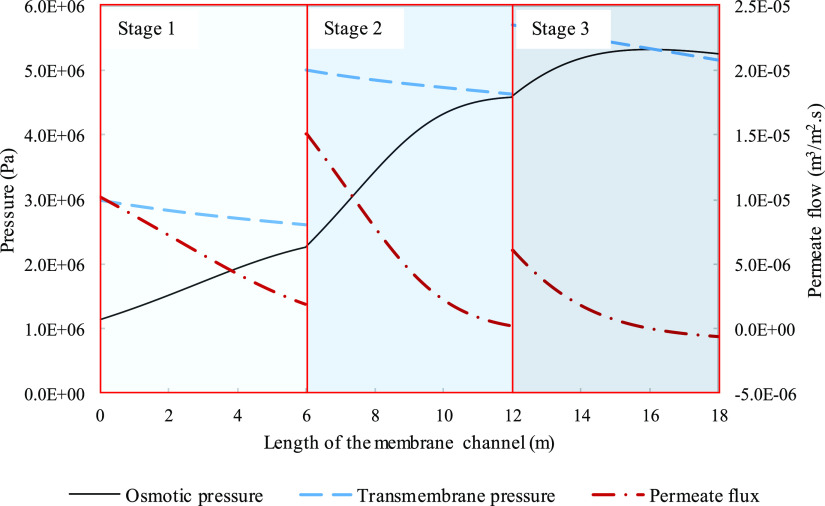
Assessing thermodynamic restriction through osmotic pressure (solid line), transmembrane pressure (dashed line), and permeate flux (dash dot line) of a three-stage system and variation along three 6 m membrane channels. Parameters used to model these values were taken from Table 3 except for pressure. Pressures for the three stages were 29.8, 50, and 57 bar in the order of appearance.

**Figure 15:**
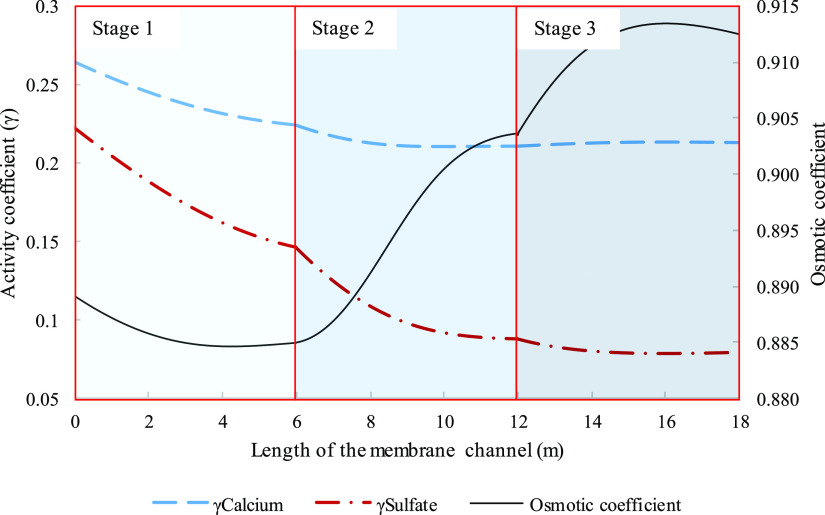
Activity coefficient of calcium (dashed line), activity coefficient of sulfate (dashed-dot line), and osmotic coefficient (solid line) were plotted of a three-stage system and variation along three 6 m membrane channels. Parameters used to model these values were taken from Table 3 except for pressure. Pressures for the three stages were 29.8, 50, and 57 bar in the order of appearance.

**Figure 16:**
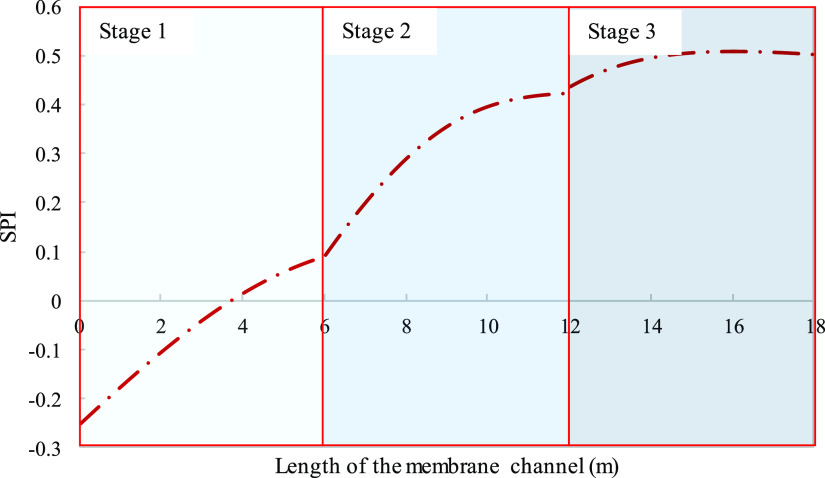
SPI (dashed-dot line) and permeate flux (solid line) were plotted of a three-stage system and variation along three 6 m membrane channels. Parameters used to model these values were taken from Table 3 except for pressure. Pressures for the three stages were 29.8, 50, and 57 bar in the order of appearance.

Brackish and seawater RO desalination typically undergoes one of the following configurations: single-stage moderate recovery or multistage overall high recovery. This is carried out to avoid the persistent issue of fouling and excessive flux decline. Figures 10–16 represent performance and water chemistry behavior under these configurations.

Figure 10 exhibits basic indicators of performance and flow behavior under the conditions of moderate product recovery. The nearly linear trends observed for the osmotic pressure and permeate flux imply that the system is only mass transfer limited. The thermodynamic parameter was modeled, in Figure 11, to showcase activity and osmotic coefficient development in a medium ionic strength environment. In Figure 11a,b, the drop by the end of the 6 m channel was 5% for both sodium and chloride and the drop was more significant at 15 and 34% for calcium and sulfate, respectively. The osmotic coefficient shows a drop, for which the reasoning behind has been discussed for Figure 4, and has a minimum of 0.8466 and an overall decrease of 0.5%. The scaling potential is also evaluated in Figure 12 where positive values are noticed past 3.7 m, far from noticeable permeate flux changes.

Figures 13–16 were modeled to achieve a maximum recovery of 0.5 at each stage and an overall recovery of 0.78, similar to that of the main case single-stage high recovery system depicted in Figure 3. It can be seen from Figure 13 that it took three stages with the last stage having a recovery of 0.13. A gradual transition from a mass transfer limited to a thermodynamically restricted system can be observed in Figure 14. The osmotic pressure started off with nearly linear behavior in stage 1 and curved toward the end of stage 2. By four meters into the third stage, the concentration level has reached thermodynamic equilibrium and the permeate reduced further to negative values, as discussed for Figure 3. The higher pressure led to a more significant pressure drop causing the overlap between osmotic pressure and the transmembrane pressure. The behavior in Figures 4 and 6b seem to be expanded over the three stages in Figure 15, achieving a similar trend of drop and increase by the end of the last stage. The irregular behavior in calcium ions where a slight increase can be viewed in the last stage has been observed elsewhere when complexed with chloride ions.^32^ The explanation for the overall trend in the osmotic coefficient has been explained prior for Figure 4. For SPI, only the first stage was partly subsaturated, while the remaining of stage and the two final stages were all operating under supersaturated conditions.

### 2.4 RO Pilot Plant Study

A pilot plant consisting of a set of pretreatment units and two membrane passes was built to assess the viability of commercializing an RO desalination plant in Dubai, UAE. Feed water composition and operation conditions were provided by the plant operators to test the capabilities of our full-scale model. A full description of the simulated RO pilot plant is included in Supporting Information section 4. The section of the plant being simulated is the first pass consisting of two pressure vessels containing two spiral wound units each to form an assembly of a 4 m long RO membrane (Figure S1). A sensitivity analysis of plant performance was implemented by changing one operating parameter at a time in the model.

#### 2.4.1 Sensitivity Analysis of Operating Parameters

A reliable model serves as a valuable tool to explore the effects of various operating scenarios at low risk to process equipment. The model presented in this study was used to perform a set of single-parameter sensitivity analyses to check the response of four system parameters: permeate flux, average permeate flux, SPI, and recovery, against changes in two input parameters: crossflow velocity and applied pressure. The permeate flux (*v*) is the volumetric rate of permeation through the membrane layer area and a metric used to assess the efficacy of clean water production (eq S.6). The average permeate flux (*V*) is the mean permeate production up to a given point *n* where *u* is the crossflow velocity and *H* is the channel height (eq 2).^8^
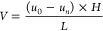
2

SPI used to assess fouling has been introduced in Section 2.2.3. The term “recovery” or “*R*” (eq 3) is an important indicator of system efficiency and refers to the following ratio
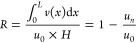
3

**Figure 17:**
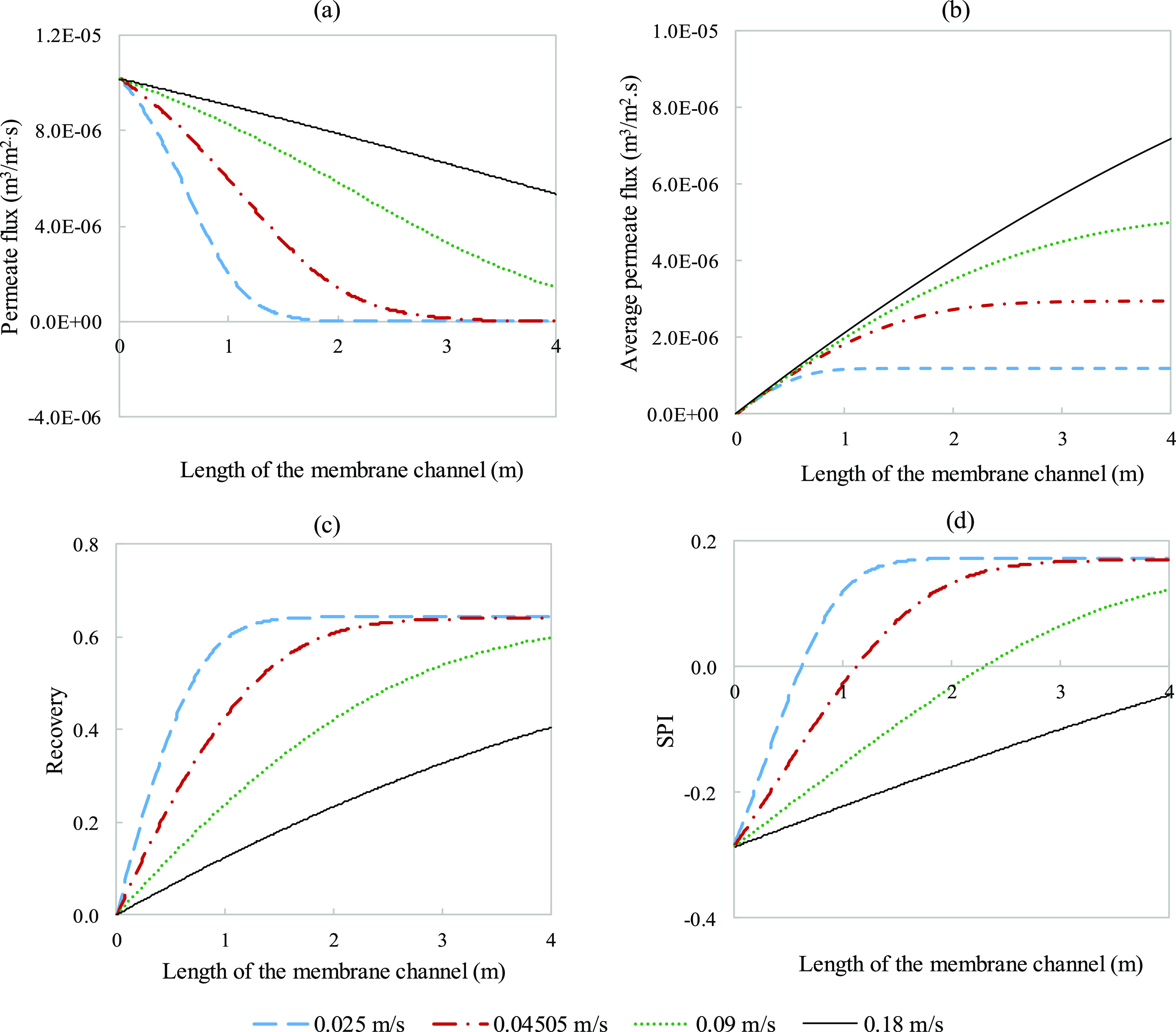
Response of main parameters (a) permeate flux, (b) average permeate flux, (c) recovery, and (d) SPI to variations in crossflow velocity plotted along the 4 m channel length. All other provided parameters used were kept the same and were taken from Tables S3 and S4.

**Figure 18:**
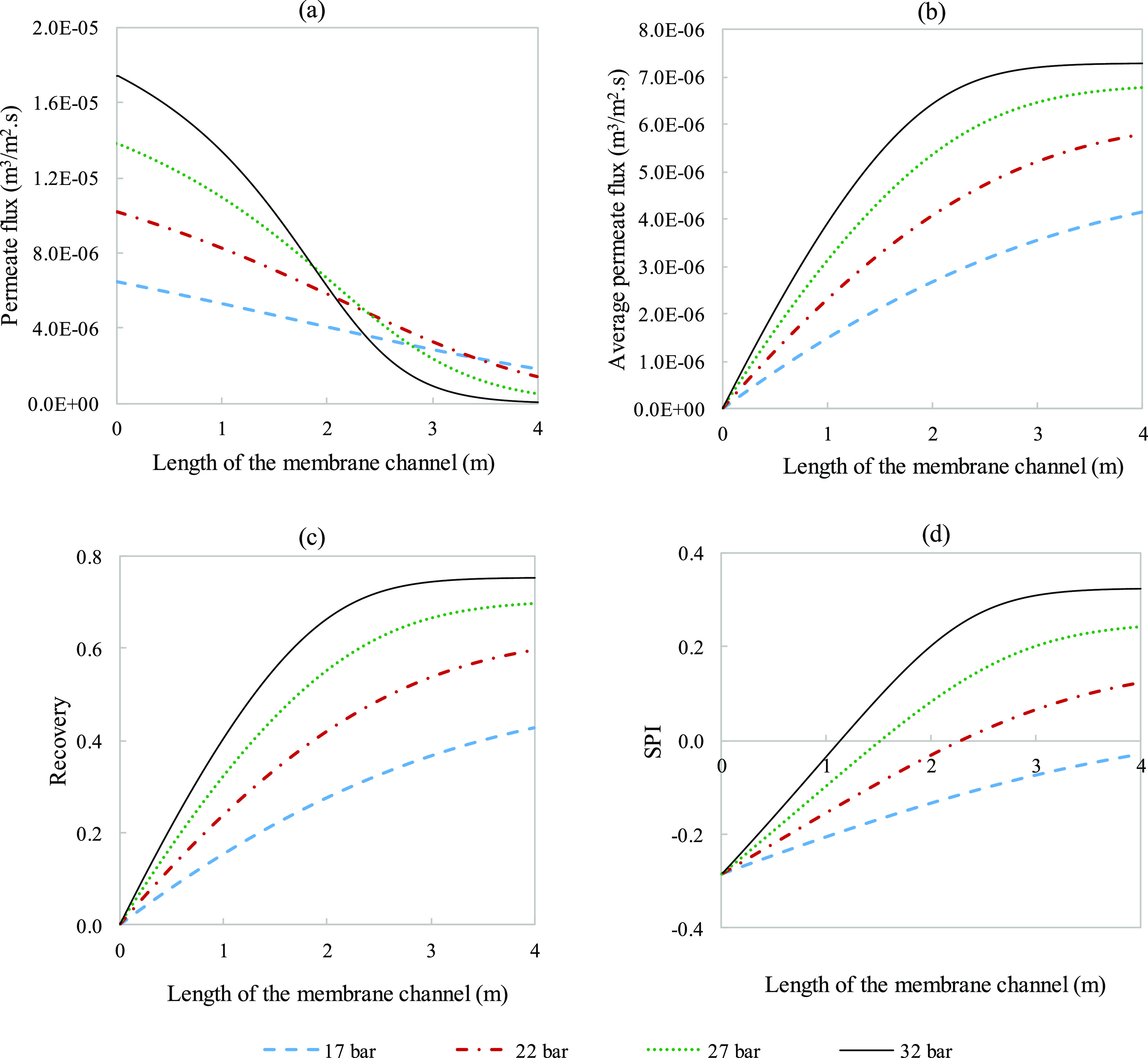
Response of main parameters (a) permeate flux, (b) average permeate flux, (c) recovery, and (d) SPI to variations in applied pressure plotted along the 4 m channel length. All other provided parameters used were kept the same and were taken from Tables S3 and S4.

The base case for the sensitivity analyses considered the following input parameter values: applied pressure at 22 bar and crossflow velocity at 0.0901 m/s. The results presented in Figure 17 deal with changing crossflow velocity, whereas in Figure 18, they deal with changing applied pressure.

**Figure 19:**
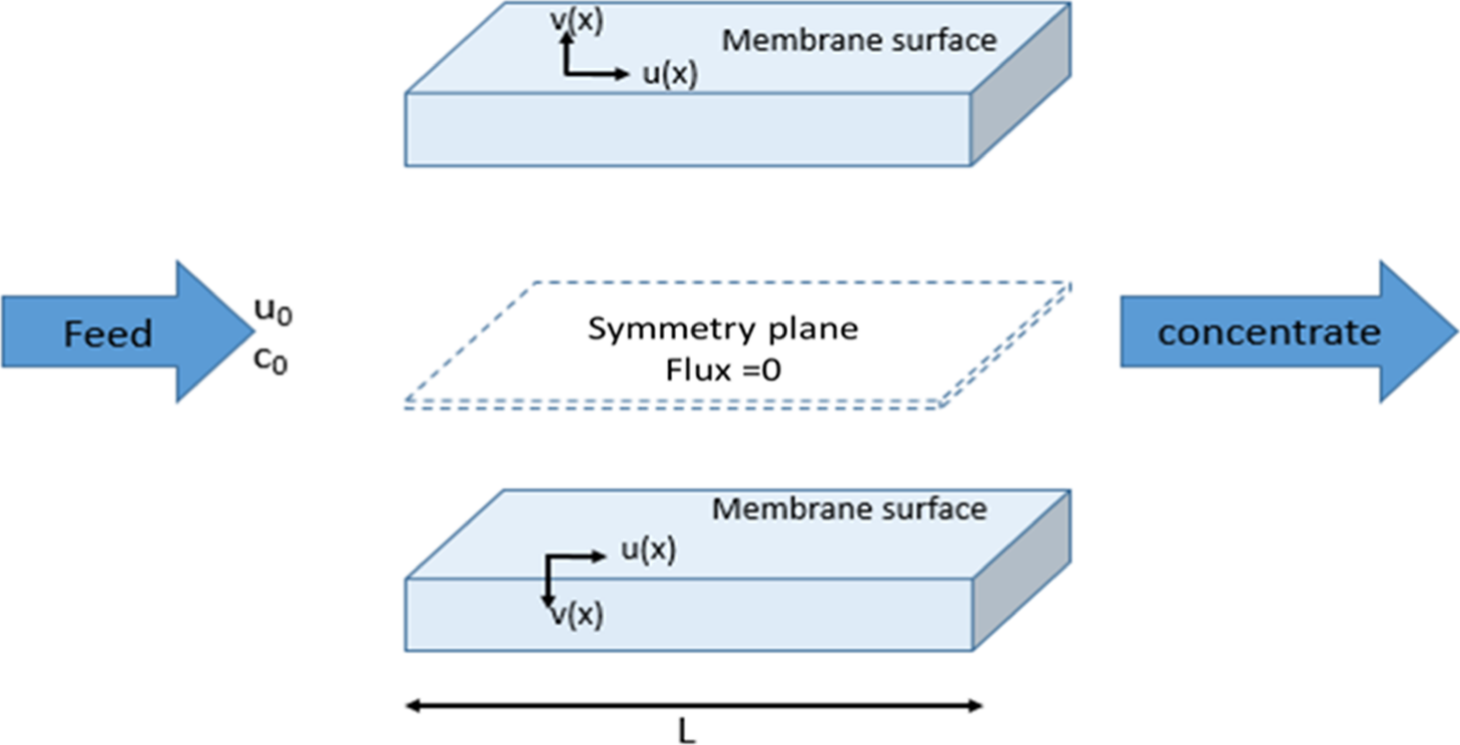
Illustration of a real RO channel comprising two parallel membranes and a feed spacer within (spacer drawing was omitted for simplicity). A symmetry plane lies in the middle of the channel. The arrows placed on the membrane represent the crossflow direction [*u*(*x*)] and the direction of permeation [*v*(*x*)].

Visualizing the effect of operational parameters on the internal performance of a filtration channel allows for process optimization and the best utilization of a membrane unit. An increase in initial crossflow velocity impedes the concentration of ions, as derived in ref (8) and shown in Supporting Information section 1. At the lowest crossflow velocity of 0.025 m/s, the membrane permeates until 1.8 m of the channel length, whereas at the highest crossflow velocity of 0.18 m/s, permeation continues throughout the whole length of the channel (Figure 17a). Both the average permeate flux and the recovery were plotted to assess the efficiency and productivity at the described run parameters. From a recovery standpoint (Figure 17c), the system achieved maximum efficiency (∼0.64) at the lowest velocity in the first meter of the channel. The figure allows us to visualize within the channel how lowering flowrate comes at the expense of production. Figure 17b highlights the trade-off between recovery efficiency and overall plant productivity. Naturally, reducing the velocity increases residence time in the membrane. This increased residence time causes the rapid increase in the brine ion concentrations, associated with accelerated fouling. For a high fouling potential feed, it is likely to be more beneficial to run at higher velocities (with lower recoveries), as implemented in practice according to ref (38).

Figure 18 assesses the effect of applied pressure on the same set of parameters. It is clear that the applied pressure directly impacts the initial rate of permeation. Additionally, the decay of the permeation flux was also affected, shifting the point of thermodynamic equilibrium further back along the membrane channel at higher pressures. Unlike the trend with velocity, the average permeate flux and recovery exhibit the same development through the channel as both improve with greater pressures. SPI values indicate that operating at lower pressures is better to mitigate fouling, which is expected from increasing the driving force that leads to a rapid increase in concentration. From low to high pressures, excluding the lowest pressure which seems to not have fouled, the distance to positive SPI decreased from 2.3 to 1.2 m (Figure 18d). The solution concentrates increase the level of the precipitating ions (calcium and sulfate) and nonprecipitating ions (sodium, chloride, and magnesium). Although the high salinity from nonprecipitating ions delay the reaction (reflected in reduced ion activity), their effect is minor in comparison to the presence of excess precipitating ions.^41^

From the observations made for the system response to changes in pressure and velocity, the significance of full-scale length channel spatial modeling can be deduced.

## 3 Conclusions and Future Work

A model was developed to cover the industrial need for a reliable computationally inexpensive predictive tool for plant-scale membrane performance based on comprehensive thermodynamic and mass transfer theoretical equations. The focus of RO models in the published literature is the fouling phenomena, reasonably so, as it intensely jeopardizes the sustainability of the process. However, rigorous thermodynamic modeling of water composition plays a significant role in other performance aspects such as the plant efficiency, separation capability, and permeate quality. The sevenfold increase in salinity by the end of a 6 m channel shows that water quality evolves rapidly and acutely in a long filtration channel and necessitates the spatial evaluation of thermodynamic properties. When evaluating the conditions described in Table 3, the model shows that by accounting for osmotic coefficient variation, the operator has a better estimation of osmotic pressure which deviates by 9% from ideality at high recovery. Value decline of up to 65% was predicted by modeling the concentration and activity of four ions (sodium, chloride, calcium, and sulfate). This value drops for a single stage with moderate recovery to 34%, which remains significant. These results will vary based on plant run parameters and feed.

**Table 3:** Parameter Values Used for Model Simulations in This Study

description	value
length of the RO system, *L* (m)	6
channel height, *H* (m)	7 × 10^–4^
applied (pump) pressure, *p*_0_ (Pa)	5.516 × 10^6^
feed salinity (mg/L)	15000
crossflow velocity at entrance, *u*_0_ (m/s)	0.2
membrane intrinsic resistance, *r*_m_ (Pa s/m)	1.8 × 1011
number of discrete spatial elements along RO channel	300
temperature, *T* (°C)	25
water viscosity at 25 °C, v (Pa s)	0.92 × 10–3
friction coefficient due to spacers	5
initial Na^+^ concentration, ppm	3450
initial Cl–, ppm	9318
initial Ca2+, ppm	658
initial SO42–, ppm	1675

Fouling potential is another crucial aspect investigated in this study as the pretreatment design depends on it. The added intricacy from spatial variation, the change in required dosage based on fluid properties in the axial direction, provide further incentive to invest in a model such as the one described. Using activity in place of concentration in fouling calculations impacted the predicted location for the onset and continuation of membrane fouling by a shift of 1 m in channel length and an overestimation by an order of a magnitude. Another key finding from the model was the large difference observed from relying on permeate flux decline rather than a reliable fouling index (in this study, SPI). In the system modeled, expected fouling starts 3 m before the point of thermodynamic equilibrium. It is suspected that the stability observed in the average permeate flux of full-scale systems during initial fouling stages as reported in the literature is due to the fact that nonpermeating zones (the region of membrane that is under thermodynamic equilibrium conditions) will be activated as earlier sections of the membrane deactivate due to the fouling accumulation, and thus, a decrease in average permeate flux will be just noted after the entire channel is exposed to severe fouling.

Data from a pilot plant were used to provide the model with a realistic input. A sensitivity analysis allowed us to envision the effects of changing applied pressure or crossflow velocity on a plant-scale system. Further validation with observed plant data and the integration of induction time for different scalants will improve the certainty of fouling prediction accuracy. Moreover, expanding model application to nanofiltration will also provide a great benefit for operators utilizing it for RO pretreatment, wastewater treatment, and numerous other purposes.

## 4 Materials and Methods

### 4.1 Model Development

The developed model, implemented in MATLAB, is used for predicting the change in concentrate composition along the membrane filtration channel. The thermodynamic modeling of RO concentrates is accomplished using the Pitzer model to calculate ionic activity coefficients (γ) and the osmotic pressure coefficient (φ) based on the predicted local concentrate compositions. The related mass transfer and thermodynamic equations are solved numerically to account for the simultaneous changes in ionic concentrations along the membrane channel and the changes in water composition. The activity model was adapted from ref (42). This model is designed to enable one to account for the activity coefficients of all ionic species present in the concentrate. The changes in concentrate density and viscosity are calculated and taken into account using the extended equation-of-state for seawater at elevated temperature and salinity.^43^ The diffusivity of each ion was accounted for through their ionic radius (found in Table S2) and varies along the membrane with viscosity as depicted in eq S.4.^44,45^ The accuracy of the activity simulation has been validated thoroughly via a comparison between the calculated data and published data through application of the same operating conditions. The transport model was verified through an experimental procedure.

#### 4.1.1 Modeling for Variation of Concentration along a Membrane Channel

A function describing the concentrations of solutes along each segment of the membrane channel was used. This was carried out in order to compute the related changes in local solute ionic and osmotic coefficients. The (full-scale) RO feed membrane channel is described as in ref (46) and visually depicted in Figure 19. Transport and conservation equations are simplified due to the symmetry plane in the middle, allowing the channel to be solved as a half channel with zero flux at the symmetrical boundary.

The full-scale RO transport model of Song et al.^5^ is modified in this study by integrating the Pitzer model to be able to account for the changes in ion activity and osmotic coefficient along defined segments in the membrane channel. The membrane transport and continuity equations used in this model are similar to those used by Song et al.^5,8,12^ to obtain the variations of concentrations along the membrane feed channel. These equations are described in Supporting Information section 1. Due to the narrow channel height, complete transverse mixing in the filtration channel is assumed, which allows the prediction to be representative of the changes along the axial direction. The boundary layer model was used to express the concentration polarization within the channel.^47^ The concentration near the wall was also deemed to be representative of species concentration in the channel as the case of complete depolarization was confirmed. It is important to note that the permeate side pressure is assumed to be neglected which allows it to be a single-dimension model.^15^

The local ion concentrations can be determined through solving eqs S.1–S.8. These equations are initially solved for the first spatial element by choosing salinity or total dissolved solids as a representative parameter for the various species present in the feed. Salinity was primarily used to predict the permeate velocity, crossflow velocity, and transmembrane pressure followed by the computation of change for each ion concentration.

#### 4.1.2 Activity Coefficient Models

Activity coefficient prediction for individual ionic species is challenging for ion-selective membranes but remains vital for the electrochemical potential calculation which dictates the thermodynamic equilibrium.^48^ Hence, the accurate prediction of scaling potential is dependent on the reliable assessment of activity coefficients.^49^ Numerous semiempirical models exist in the literature based on the Poisson–Boltzmann equation and the Debye–Hückel theory for the assessment of thermodynamic parameters. Commonly used models are discussed in ref.^49,50^ Pitzer et al. developed the most broadly used set of equations and has discussed them thoroughly.^50−55^

### 4.2 Debye–Hückel Theory

The Debye–Hückel theory takes into account ions as charged species with a constant ionic diameter. This limited its use to dilute solutions 0.001 *m* (molal) which led to the development of the extended Debye–Hückel theory. The extended form considered the ionic diameter of different species and allowed for its application for solutions up to 0.1 *m*. Further details are included in Supporting Information section 2.

Pitzer built on these the Debye–Hückel theories^51,55^ and widened their applicability to high concentrations up to 6 *m*. The next passage will provide a description of the Pitzer equations used in this study.

### 4.3 Pitzer Model

Pitzer overcame the limitation of describing high ionic strength solutions in the Debye–Huckel equations by incorporating the interaction parameters between ionic species. Equation 4 represents the base Pitzer formulation defining total excess Gibbs energy.
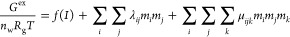
4

Starting from left to right, *f*(*I*) describes the Debye–Huckel portion of the equation and is dependent on the ionic strength and the solvent dielectric constant. The two remaining terms express the binary and ternary interaction parameters. *i, j*, and *k* subscripts represent different cations and anions; *m*_*i*_ is molality (moles per kilogram); and *n*_w_ is the number of kilograms of water. λ_*ij*_ is the second virial coefficient which considers the short-range forces between two species and is dependent on the ionic strength. μ_*ijk*_ is the third virial coefficient and accounts for these forces among three species and is independent of the ionic strength.^54^

From derivations of eq 4, the activity coefficient (γ) equations were created. Defined in eqs 5 and 6 as follows
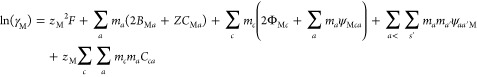
5
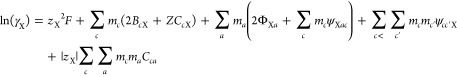
6

Solvent nonideality is expressed through the osmotic coefficient (ϕ_osmotic_) in eq 4 and takes part in the prediction of osmotic pressure in the RO channel.
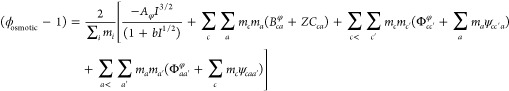
7

The M, *c*, and *c*′ subscripts represent cations, while X, *a*, and *a*′ subscripts represent anions in eqs 5–7. M and X indicate ions of interest; *c* and *a* are summation indices of all cations and anions, respectively; *c*′ and *a*′ are summation indices for different ions. Terms *F*, *B*, *C*, Φ, and ψ denote parameters used for the calculation of the second and third virial coefficients λ and μ. All model-incorporated Pitzer parameters mentioned in this section and more are further described in Supporting Information section 2.

### 4.4 Model Algorithm

**Figure 20:**
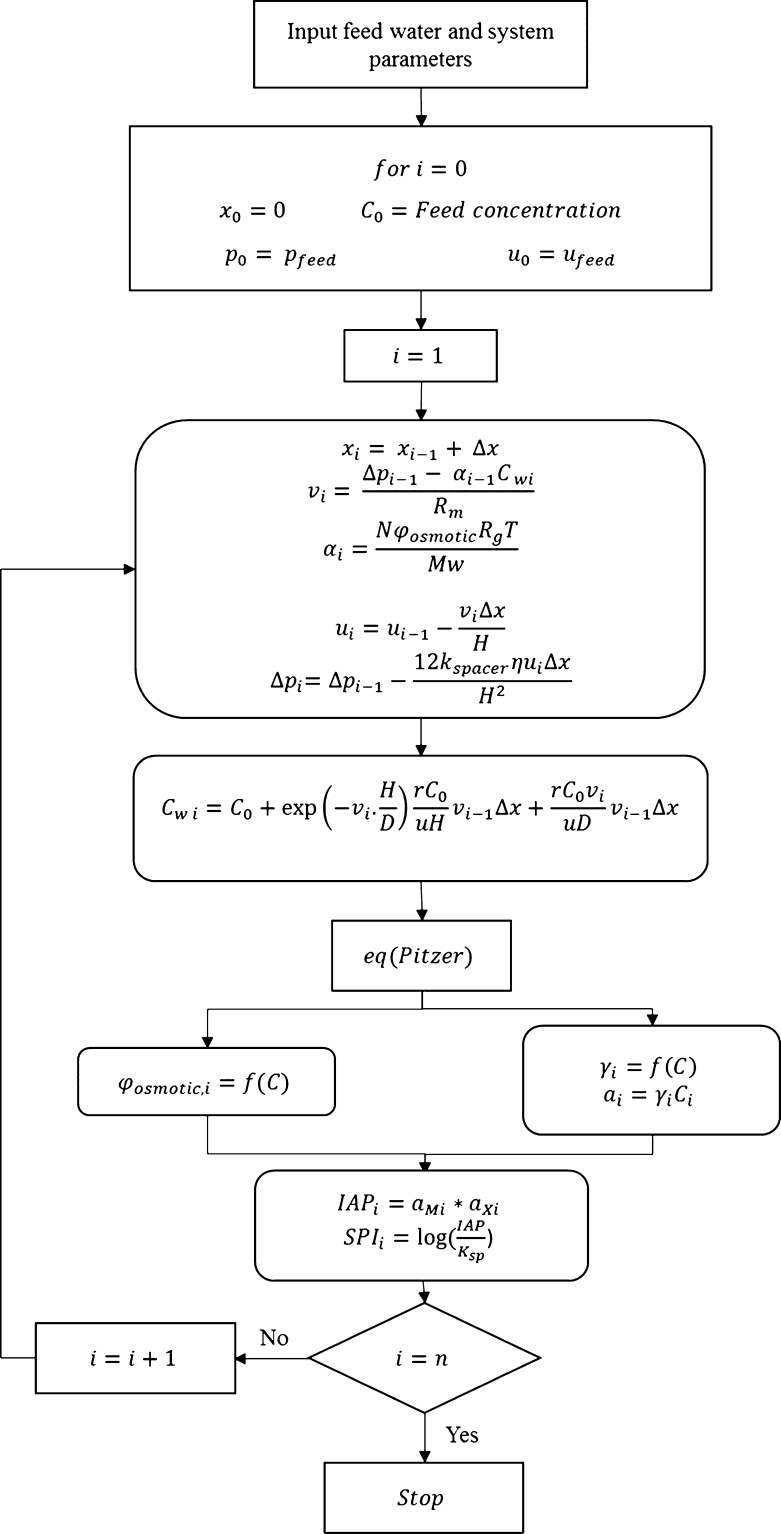
Algorithm for solving the mathematical model.

In order to achieve a thermodynamic modeling of saline waters, a MATLAB-based implementation of Pitzer (eqs 4–7, S.12–S.30) and mass transfer models was written to determine the thermodynamic changes in the brine concentrate using a discretized (finite-element) approach. The details of step-size determination for discretization can be found by the model creator elsewhere.^27^ The long membrane filtration channel was divided into small spatial segments in the axial direction. A recursive algorithm was used to solve for the hydrodynamic and thermodynamic parameters for each spatial segment. Figure 20 shows the algorithm that has been used in this simulation. The simulation parameters can be chosen for given specific operating conditions and from the manufacturers’ specifications for modules. Parameters shown in Table 3 serve as an example.

### 4.5 Experimental Validation

**Figure 21:**
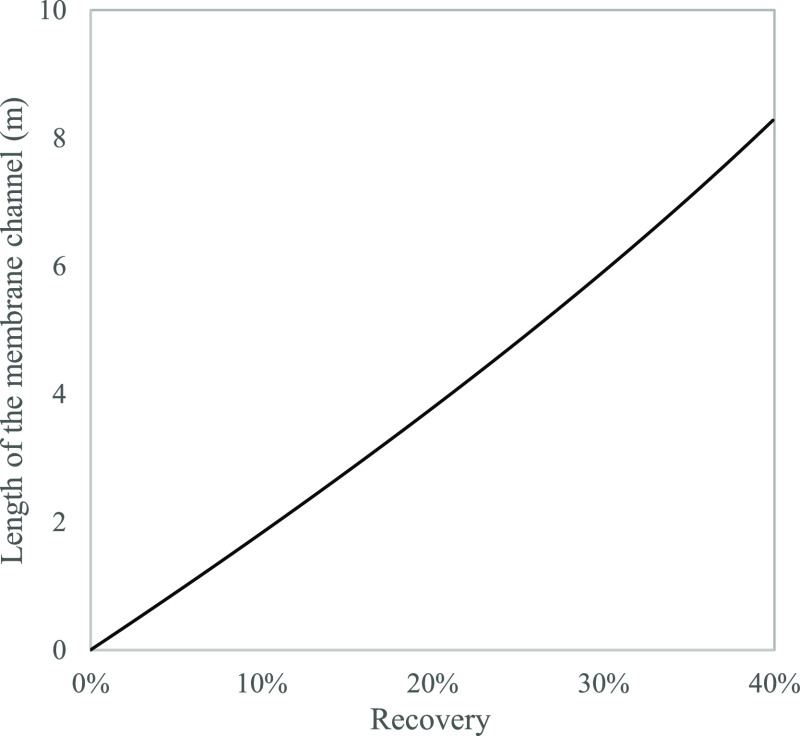
Length of the membrane channel required as a function of recovery.

Drak et al.^56^ developed a technique involving an intermittent recycle flow regime for bench-scale systems where a relationship was established between small- and full-scale systems through product recovery. Successful prediction was reported for the field results of three out of four pilot plants using this technique. This method was applied to validate the model accuracy in simulating full-scale concentrate behavior by relating recovery to channel length (Figure 21).

**Figure 22:**
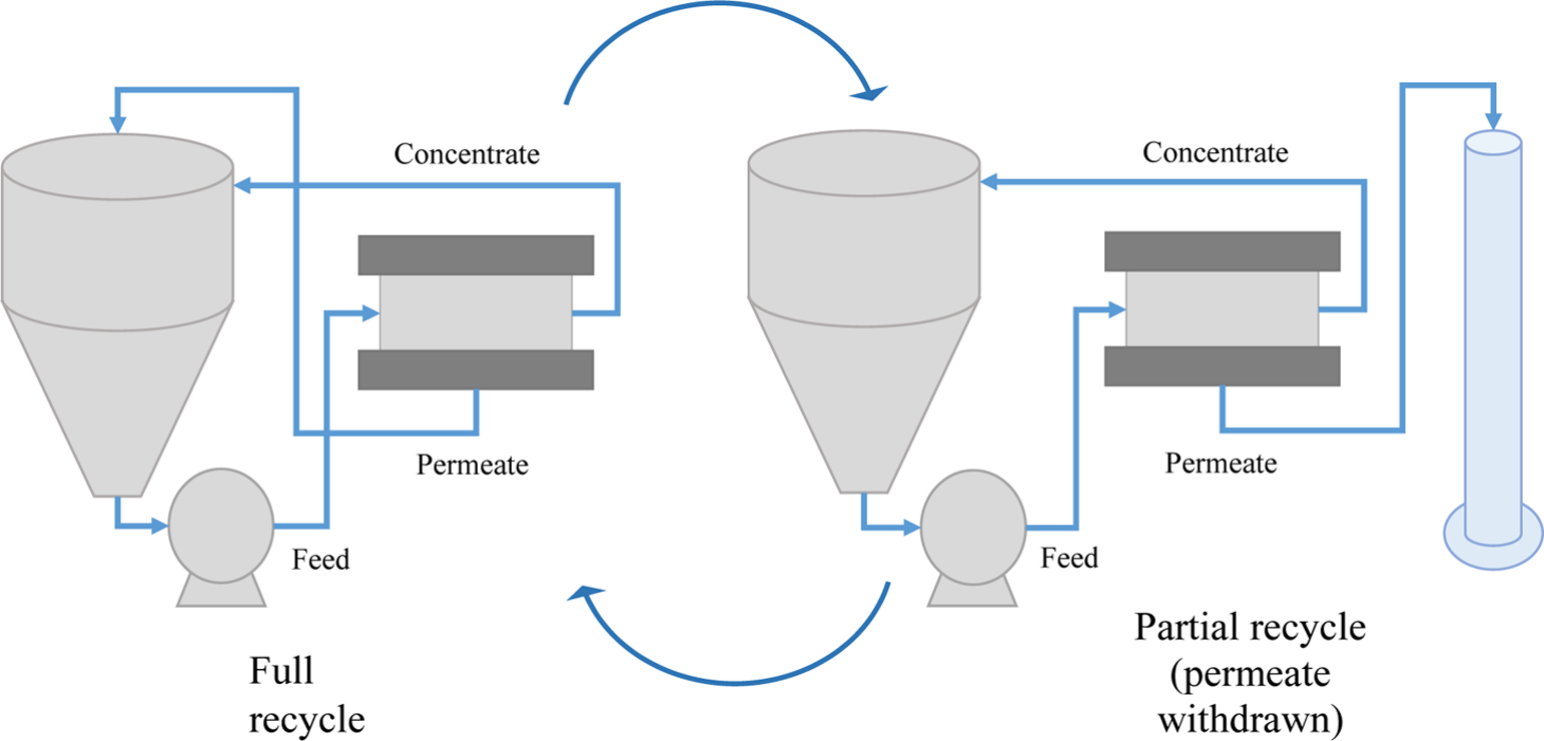
Visual representation of the intermittent flow recycle regime applied here for experimental validation. The system is run in alternating modes of full recycle (both the permeate and concentrate are recycled) maintaining concentrate salinity and partial recycle (only the concentrate is recycled and pure water is removed) allowing for salinity increase to imitate solution behavior in a long filtration channel.

The setup comprised a standard membrane rig that includes a high-pressure pump and a feed tank with a crossflow cell (Sterlitech, US). A 1% saline solution was prepared by dissolving NaCl (Honeywell, Germany) and distilled water with a conductivity of <10 μS/cm (GFL, Switzerland). Based on the feed, a brackish water flat sheet membrane BW30XFR (Filmtec, US) was selected. Lab-prepared calibration solutions were made by dissolving reagent-grade NaCl in type I deionized Purite water (Suez, France). A graduated cylinder along with a digital mass balance was used to quantify the permeate. The solution was run through the system at 29 bar, 25 °C with a flow rate of 4.1 L/min. After allowing adequate time for system pressure and product flow stabilization, the initial concentrate water sample was taken and measured for conductivity. During the sampling, the flow rate was also measured in the permeate line by manually collecting water for a minute and measuring its mass. This was carried out in triplicates for each sample. This is followed by the removal of 10% of solution volume as permeate to represent 10% recovery and then re-establishing the full-recycle mode (Figure 22). Measurements of flow and conductivity were taken after 2 h of stabilization expecting an increased level of salinity (*y*_1@10%_). The process of permeate removal, stabilization, and measurement was repeated until the desired recovery level was reached.

Solution conductance and mass measurements were used as quantifiable properties for ion concentration and product flow rate. As electrochemical devices measure solution activity rather than concentration,^57^ a correction factor in the form of the water activity coefficient along with lab-prepared standard NaCl solutions was used in order to compare concentration prediction.

Water activity (*a*_w_) accounts for solvation effects and solute–solvent interactions and is calculated through eq 8.^58,59^
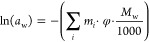
8

It connects to the previously described osmotic coefficient (φ) and takes the molality of the ionic species present (*m*_*i*_) and the molar mass of water (*M*_w_) as inputs.

## Supporting Information Available

Governing equations for mass transfer, extensive Pitzer model equations, equations for fouling prediction, description of the brackish water RO pilot plant system, and additional run parameters (PDF)

## Nomenclature

*A*_Φ_: interaction parameter Pitzer model (−)
*B*,*B*′, *B*^Φ^: interaction parameter Pitzer model (−)
*c*_0_: initial feed salt concentration, mg/L
*c*(*x*): localized feed salt concentration, mg/L
*c*_w_(*x*): localized salt concentration on the membrane surface, mg/L
*C*,*C*^Φ^: interaction parameter Pitzer model (−)
*D*: diffusion coefficient, m^2^/s
*f*^Φ^: interaction parameter Pitzer model (−)
*G*: standard Gibbs free energy
*I*: ionic strength, mol/Kg
*K*_sp_: thermodynamic solubility product
*M*_w_: molecular weight of the solute
*n*: number of segments
*N*: the number of ions in solution that can result from one salt molecule
*P*: process pressure, bar
*P*_0_: reference pressure, 1 bar
*p*_f_: the static pressure in the feed, Pa
*p*_p_: the static pressure in the permeate, Pa
*R*_g_: the universal gas constant
*R*_m_: membrane resistance, Pa s/m
*T*: temperature, K
*t*: time, s
*v*: permeate velocity, m/s
*v*(*x*): localized permeate flux, m/s
*z*_*i*_: ionic charge of component *i* (−)
*Z*: modified ionic strength, mol/Kg
**Greek letters**
α: osmotic coefficient, Pa L/mg
α_1_,α_2_: interaction parameter Pitzer model (−)
Δ: the difference between two values
Δ*c*(x): localized difference between feed salt concentration and permeate salt concentration
Δ*c*_w_(x): localized difference between feed salt concentration on the membrane surface and permeate salt concentration
Δπ(*x*): localized osmotic pressure across the membrane, Pa
Δ_f_*G*^Φ^: standard Gibbs free energy change of formation
Δ_reac_*G*^Φ^: standard molar Gibbs free energy change of reaction
Δ*H*_reac_: enthalpy change of reaction
Δ_r_*H*°: standard enthalpy change of the precipitation reaction
Δ*p*: the transmembrane static pressure, Pa
Δ*p*(x): localized transmembrane static pressure, Pa
Δ*p*_0_: initial transmembrane pressure, Pa
Δ*p*_fric_: frictional losses due to friction by the membrane surfaces and spacers
γ: activity coefficient
ρ: density, Kg/m3
η: water viscosity, Pa s
Φ,Φ^’^,Φ^Φ^: interaction parameter Pitzer model (−)
Φ_osmotic_: Pitzer osmotic coefficient
λ_*ij*_: the second virial coefficient
μ_*ijk*_: third virial coefficient
Ψ: interaction parameter Pitzer model (−)
ξ: dummy integration variable
**Subscripts**
0: reference or initial value
*I*: segment number or component index
*J*: component index
*N*: *n*th component
*W*: the variable at the membrane wall
**Superscripts**
Φ: reference temperature value
0: reference value
*f*: formation
*r*: reaction
**Abbreviations**
IAP: ion activity product
LPF: local permeate flux
NF: nanofiltration
RO: reverse osmosis
SPI: scaling potential index
*s*: solid phase
