# Supplementary material for: Robust Spatial Modeling of Thermodynamic Parameters in a Full-Scale Reverse Osmosis Membrane Channel
Source: ACS Omega. 2021 May 7;6(19):12392–409. doi: 10.1021/acsomega.0c04412 (PMC8154145; doi:10.1021/acsomega.0c04412)
Supplement: Supplementary file 1 — ao0c04412_si_001.pdf [file ao0c04412_si_001.pdf]

SUPPORTING INFORMATION

A Robust Spatial Modelling of Thermodynamic Parameters in Full-Scale Reverse Osmosis Membrane Channel

Afra Alkatheeri[a] , Ramis Rafay[a] , Emad Alhseinat[a,b*,] Ahmad Safieh[c] , Fadi Alnaimat[d]

[a] Department of Chemical Engineering, Khalifa University of Science and Technology, Abu Dhabi, United Arab Emirates

[b] Center for Advanced Membranes and Water Technologies, Abu Dhabi, United Arab Emirates

[c] Research & Development Department, Dubai Electricity & Water Authority (DEWA), Dubai, United Arab Emirates

[d] Mechanical Engineering Department, United Arab Emirates University (UAEU), Al Ain, United Arab Emirates.

S 1. Concentration variation model

Table S 1 – Governing equations for the modelling of concentration variation in RO long membrane channel adapted from articles by Song et al. [1,2].

	Parameter	Equation	Variable definition
S.1	Cross flow velocity	$u(x) = u_0 - \frac{1}{H}\int_0^x v(\xi)d\xi$	$u(x)$: cross flow velocity at location x<br>H: height of the membrane channel<br>u_0: feed flow velocity<br>v: permeate flux<br>ξ: dummy integration variable
S.2	Bulk concentration	$c(x) = \frac{1}{u(x)H}\left[c_0 u_0 H - (1-r)\int_0^x c(\xi)v(\xi)d\xi\right]$	$c(x)$: concentration of any given species at location x<br>c_0: species concentration at the feed channel entrance<br>r: fraction of the species rejected by the membrane
S.3	Concentration near the wall	$c_w(x) = c_0 + \exp\left(-\frac{v(x)H}{D}\right)\frac{rc_0}{uH}\int_0^x v(\xi)d\xi + \frac{rc_0 v(x)}{uD}\int_0^x v(\xi)d\xi$	$c_w(x)$: concentration on the membrane surface or membrane wall at point x<br>D: dispersion coefficient with empirical constant (c=8) dependent on spacer properties[2]
S.4	Hydraulic dispersion coefficient	$D = 8d_m = \frac{8k_bT}{6\pi\mu(T,S)a_i}$	D: dispersion coefficient with empirical constant (c=8) dependent on spacer properties[2]<br>d_m: diffusivity of each species

			k_b: Boltzmann's constant<br>T: absolute temperature<br>μ: dynamic viscosity dependent on salinity and temperature<br>a_i: ionic radius of each species
S.5	Transmembrane static pressure	$\Delta p(x) = \Delta p_0 - \left[\frac{12 k_{spacer}\eta}{H^2}\int_0^x u(\xi)d\xi\right]$	Δp: difference between static pressure in the feed channel and the permeate channel<br>k_{spacer}: friction coefficient due to the feed spacer<br>η: dynamic viscosity
S.6	Permeate flux	$v(x) = \frac{p(x) - \Delta\pi(x)}{R_m}$	$v(x)$: permeate flux at location x<br>R_m: membrane resistance to water passage<br>$\Delta\pi$: osmotic pressure difference
S.7	Osmotic pressure difference	$\Delta\pi(x) = \alpha\Delta c(x)$	α: coefficient that relates the osmotic pressure to concentration
S.8	Osmotic coefficient	$\alpha = \frac{N\phi_{osmotic}R_g T}{MW}$	N: number of ions in solution that can result from one salt molecule (N is 2 for NaCl)<br>R_g: universal gas constant<br>T: absolute temperature<br>MW: molecular weight of the solute<br>$\phi_{osmotic}$:Pitzer osmotic coefficient.

Table S 2 – Ionic radii used for the diffusivity calculation for the present ionic species. Values were taken from [3].

Cation	Ion radius (m)	Anion	Ion radius (m)
H	1.30×10^{-10}	Cl	1.68×10^{-10}
Ca	1.00×10^{-10}	SO_4	2.18×10^{-10}
Mg	7.20×10^{-11}	HSO_4	2.21×10^{-10}
Na	1.02×10^{-10}	HCO_3	2.07×10^{-10}
K	1.38×10^{-10}	CO_3	1.89×10^{-10}
Sr	1.13×10^{-10}	OH	1.52×10^{-10}
Ba	1.36×10^{-10}	-	-

S 2. Activity models

Debye-Huckel theory

Equations belonging to the Debye-Hückel theories defined in section 2.2.1 will be further described below. Activity coefficients (γ) are dependent on the ionic strength (Eq. S.9) and independent from the ionic diameter in the Debye-Hückel limiting law as expressed in Eq. S.10.

$$I = \frac{1}{2}\sum_i m_i . z_i^2 \quad \text{(S.9)}$$

$$log\, \gamma_i = -A.z_i^2.I^{1/2} \quad \text{(S.10)}$$

I representing the ionic strength; mi the molality of species I; z_i the charge of ion i; A is a parameter describing the relative permittivity of solvent and the temperature and has a value of 0.509 / (mol kg^{-1})$^{1/2}$ for aqueous solutions at 25 ^{o}C.

Eq. S.11 provides the definition of the extended Debye- Hückel theory which accounts for ionic diameters.

$$log\, \gamma_i = \frac{-A.z_i^2.I^{1/2}}{1 + b.I^{1/2}} \quad \text{(S.11)}$$

b is the distance between ions. Eq. S.11 turns to S.10 at low I values.

Pitzer model

A set of equation were written in MATLAB to model Pitzer activity and osmotic coefficients. In section 2.2.2, the main equations describing the essential excess Gibbs free energy relation, cation and ion activities, and solvent osmotic coefficient were included. The parameters linked to the binary and ternary interactions are added here to fully demonstrate the intricacy of the Pitzer thermodynamic model presented in Eqs S.12-S.30:

F: the repeated part in both the cation and anion activity coefficient calculations in Eqs. 2 and 3.

$$F = f^{\gamma} + \sum_c \sum_a m_c m_a B'_{ca} + \sum_{c<} \sum_c m_c m_c \Phi'_{cc'} + \sum_{a<} \sum_a m_a m_a \Phi'_{aa'} \qquad \text{(S.12)}$$

$$f^{\gamma} = -A_{\varphi}\left[\frac{I^{1/2}}{1 + bI^{1/2}} + \frac{2}{b} ln\left(1 + bI^{1/2}\right)\right] \qquad \text{(S.13)}$$

A_{φ}: the Debye-Hückel slope; changes with temperature [4]

b: distance between ions = 1.2 (selected by Pitzer [5])

$$A_{\varphi} = 3.6901531 \times 10^{-1} - 6.32100430 \times 10^{-4}T + \frac{9.1425359}{T} - 1.35143986 \times 10^{-2} ln\, T + \frac{2.26089788 \times 10^{-3}}{T - 263} + 1.92118597 \times 10^{-6}T^2 + \frac{4.52586464 \times 10^{1}}{680 - T} \qquad \text{(S.14)}$$

T: absolute temperature, Kelvins

B: terms that define the second virial coefficient; $B_{MX} = B_{ca}$

C: terms that describe the third virial coefficient; $C_{MX} = C_{ca}$

$$B_{MX} = \beta_{MX}^{(0)} + \beta_{MX}^{(1)} g\left(\alpha_1 I^{1/2}\right) + \beta_{MX}^{(2)} g\left(\alpha_2 I^{1/2}\right) \qquad \text{(S.15)}$$

$$g(x) = 2\left[1 - (1 - x)e^{-x}\right]/x^2 \qquad \text{(S.16)}$$

$$g'(x) = -2\left[1 - (1 + x + 0.5x^2)e^{-x}\right]/x^2 \qquad \text{(S.17)}$$

$$B'(x) = \beta_{MX}^{(1)} g'\left(\alpha_1 I^{1/2}\right)/I + \beta_{MX}^{(2)} g'\left(\alpha_2 I^{1/2}\right)/I \qquad \text{(S.18)}$$

$$B_{MX}^{\varphi} = \beta_{MX}^{(0)} + \beta_{MX}^{(1)} exp\left(-\alpha_1 I^{1/2}\right) + \beta_{MX}^{(2)} exp\left(-\alpha_2 I^{1/2}\right) \qquad \text{(S.19)}$$

$$C_{MX} = \frac{C^{\varphi}}{2|z_M z_X|^{1/2}} \qquad \text{(S.20)}$$

$$Z = \sum_i m_i |z_i| \qquad \text{(S.21)}$$

Φ: binary interactions of two-salt mixtures (cation-cation)

Ψ: ternary interactions of two-salt mixtures (cation-cation-cation)

$$\Phi_{ij}^{\varphi} = \theta_{ij} + I^{E}\theta_{ij}'(I) + {}^{E}\theta_{ij}(I) \quad \text{(S.22)}$$

${}^{E}\theta_{ij}$, ${}^{E}\theta'_{ij}$: dissimilar ion pairs (cation, cation) electrostatic mixing effects; zero when pairs have the same charge [6].

$$\Phi_{ij} = \theta_{ij} + {}^{E}\theta_{ij}(I) \quad \text{(S.23)}$$

$$\Phi_{ij}' = {}^{E}\theta_{ij}'(I) \quad \text{(S.24)}$$

$$E\theta_{ij}(I) = \left(\frac{|z_i z_j|}{4I}\right)[J(x_{ij}) - 0.5J(x_{ii}) - 0.5J(x_{jj})] \quad \text{(S.25)}$$

$${}^{E}\theta_{ij}' = \left(\frac{-{}^{E}\theta_{ij}}{I}\right) + \left(\frac{|z_i z_j|}{8I^2}\right).[x_{ij}J''(x_{ij}) - 0.5x_{ii}J''(x_{ii}) - 0.5x_{jj}J''(x_{jj})] \quad \text{(S.26)}$$

$$x_{ij} = 6|z_i z_j|A_{\varphi}I^{0.5} \quad \text{(S.27)}$$

$$J(x) = x[4 + 4.581x^{-0.7237}exp\,(-0.0120x^{0.528})]^{-1} \quad \text{(S.28)}$$

$$J''(x) = \frac{4 + [4.581x^{-0.7237}exp\,(-0.0120x^{0.528})][0.006336x^{0.528} + 1.7237]}{[4 + 4.581x^{-0.7237}exp\,(-0.012x^{0.528})]^2} \quad \text{(S.29)}$$

$$X(T) = a_1 + a_2T + \frac{a_3}{T} + a_4 ln\,T + \frac{a_5}{T-263} + a_6T^2 + \frac{a_7}{680-T} + \frac{a_8}{T-227} + a_9T^3 + a_{10}T^4 \quad \text{(S.30)}$$

X(T): Pitzer parameters $\beta^{(0)}$, $\beta^{(1)}$,$\beta^{(2)}$,θ ,Ψ , *and* C^{ϕ}

a_i: constants available in literature for different ionic pairs

S 3. Fouling prediction

Scaling potential index (SPI) was selected to predict the onset of inorganic fouling in the system with the operating parameters described in Table 3. The predicted ion activities were used to calculate the SPI at different segments of the membrane through Eq. S.31-S.33:

$$SPI = log_{10}(IAP/K_{sp}) = log_{10}(IAP/EXP(-\Delta_{reac}G^{\varphi}/R_g\,T)) \quad \text{(S.31)}$$

$$-R_gTln\,(K_{sp}) = \Delta_{reac}G^T = \frac{T}{298.15}\Delta_{reac}G^{298} + \Delta_{reac}H^{298}\left(1 - \frac{T}{298.15}\right) \quad \text{(S.32)}$$

$\Delta_{reac}G^{\Phi}$: Gibbs free energy of reaction

IAP = $\{Ca^{+2}\}\ \{SO_4^{-2}\}\ \{H_2O\}^2 = (\gamma_{Ca^{+2}}[Ca^{+2}])(\gamma_{SO4^{-2}}[SO4^{-2}])(a_{H_2O})^2$ (S.33)

IAP: ion activity product.

R_g: gas constant (1.987× 10^{-3} kcal/mol.K)

T: temperature (K)

When SPI is negative the solution has virtually no risk of precipitation fouling. While positive values indicate the likelihood of scaling in the system.

S 4. DEWA pilot plant

The DEWA reverse osmosis pilot plant consists of two passes. The first pass has two stages in series, with one pressure vessel each. The second pass has three stages in series, stage 1 consists of two pressure vessels in parallel and stages 2 and 3 consist of one pressure vessel. The first pass (Figure S1) was simulated using the water composition and feed and membrane properties provided by DEWA. The MATLAB model is a powerful tool which is able to give an indication at which axial point along the membrane module fouling will occur by considering the local thermodynamic behavior of the feed. Table S3 and S4 show the values which were used in the model.

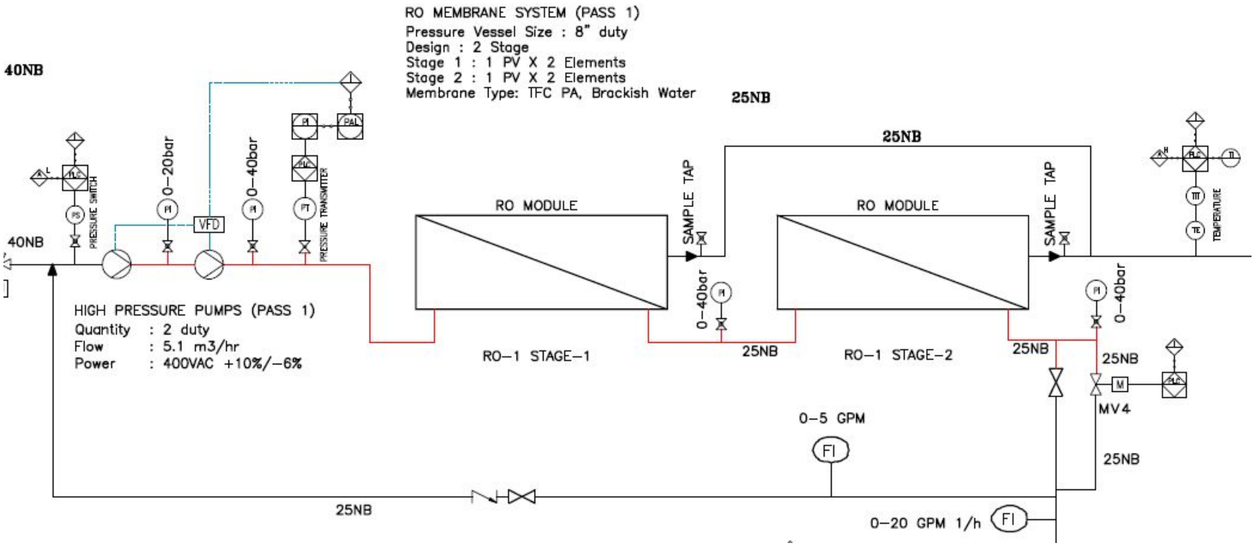

Figure S 1 – Segment of the first pass in a RO pilot plant schematic

Table S 3 – Parameters provided by the pilot plant operators

Parameter	Value
Feed temperature (°C)	35
Initial applied pressure (Pa)	2.2 x 10^6
Length (m)	4
Feed spacer thickness (mil)	34
Membrane area (m^2) *	37
Flow (m^3/h)	5.1
Salinity (mg/L)	10870
Sodium (mg/L)	3339
Magnesium (mg/L)	236.9
Calcium (mg/L)	320
Chloride (mg/L)	4180
Sulfate (mg/L)	2794.8

*Area provided is per 8 inch diameter/40 inch length spiral wound module

Table S 4 – Calculated parameters through membrane manufacturer data

Parameter	Value
Channel height (m)	0.0008636
Feed cross-flow velocity (m/s)	0.0901
Membrane intrinsic resistance (Pa s/m) *	1.37×10^{11}

*calculated through membrane manufacturer test conditions

References

[1] Song, L.; Hong, S.; Hu, J. Y.; Ong, S. L.; Ng, W. J. Simulations of Full-Scale Reverse Osmosis Membrane Process. *J. Environ. Eng.*, **2002**, *128* (10), 960–966. https://doi.org/10.1061/(asce)0733-9372(2002)128:10(960).

[2] Zhou, W.; Song, L.; Guan, T. K. A Numerical Study on Concentration Polarization and System Performance of Spiral Wound RO Membrane Modules. *J. Memb. Sci.*, **2006**, *271* (1–2), 38–46. https://doi.org/10.1016/j.memsci.2005.07.007.

[3] Marcus, Y. *Ions in Solution and Their Solvation*; 2015. https://doi.org/10.1002/9781118892336.

[4] Ller, N. M.; Møller, N. The Prediction of Mineral Solubilities in Natural Waters: A Chemical Equilibrium Model for the Na-Ca-Cl-SO4-H2O System, to High Temperature and Concentration. *Geochim. Cosmochim. Acta*, **1988**, *52* (4), 821–837. https://doi.org/10.1016/0016-7037(88)90354-7.

[5] Pitzer, K. S.; Mayorga, G. Thermodynamics of Electrolytes. II. Activity and Osmotic Coefficients for Strong Electrolytes with One or Both Ions Univalent. *J. Phys. Chem.*, **1973**, *77* (19), 2300–2308. https://doi.org/10.1021/j100638a009.

[6] Plummer, L.; Parkhurst, D.; Fleming, G.; Dunkle, S. *A Computer Program Incorporating Pitzer's Equations for Calculation of Geochemical Reactions in Brines*; **1988**. https://doi.org/10.3133/wri884153.
